# *In Situ* Microparticles Loaded with *S*-Nitrosoglutathione Protect from Stroke

**DOI:** 10.1371/journal.pone.0144659

**Published:** 2015-12-08

**Authors:** Marianne Parent, Ariane Boudier, Julien Perrin, Claude Vigneron, Philippe Maincent, Nicolas Violle, Jean-François Bisson, Isabelle Lartaud, François Dupuis

**Affiliations:** 1 CITHÉFOR EA 3452, Faculty of Pharmacy, Université de Lorraine, Nancy, France; 2 INSERM U1116, Faculty of Medicine, Université de Lorraine, Vandœuvre-lès-Nancy, France; 3 ETAP–Ethologie Appliquée, Research Centre in Pharmacology, Nutrition and Toxicology, Vandœuvre-lès-Nancy, France; INSERM U894, FRANCE

## Abstract

Treatment of stroke, especially during the first hours or days, is still lacking. *S*-nitrosoglutathione (GSNO), a cerebroprotective agent with short life time, may help if administered early with a sustain delivery while avoiding intensive reduction in blood pressure. We developed *in situ* forming implants (biocompatible biodegradable copolymer) and microparticles (same polymer and solvent emulsified with an external oily phase) of GSNO to lengthen its effects and allow cerebroprotection after a single subcutaneous administration to Wistar rats. Arterial pressure was recorded for 3 days (telemetry, n = 14), whole-blood platelet aggregation up to 13 days (aggregometry, n = 58), and neurological score, cerebral infarct size and edema volume for 2 days after obstruction of the middle cerebral artery by autologous blood clots (n = 30). GSNO-loaded formulations (30 mg/kg) induced a slighter and longer hypotension (-10 *vs*. -56 ± 6 mmHg mean arterial pressure, 18 h *vs*. 40 min) than free GSNO at the same dose. The change in pulse pressure (-50%) lasted even up to 42 h for microparticles. GSNO-loaded formulations (30 mg/kg) prevented the transient 24 h hyper-aggregability observed with free GSNO and 7.5 mg/kg-loaded formulations. When injected 2 h after stroke, GSNO-loaded microparticles (30 mg/kg) reduced neurological score at 24 (-62%) and 48 h (-75%) *vs*. empty microparticles and free GSNO 7.5 mg/kg and, compared to free GSNO, divided infarct size by 10 and edema volume by 8 at 48 h. Corresponding implants reduced infarct size and edema volume by 2.5 to 3 times. The longer (at least 2 days) but slight effects on arterial pressures show sustained delivery of GSNO-loaded formulations (30 mg/kg), which prevent transient platelet hyper-responsiveness and afford cerebroprotection against the consequences of stroke. In conclusion, *in situ* GSNO-loaded formulations are promising candidates for the treatment of stroke.

## Introduction

Treatments to protect the brain during the first days following ischemic stroke are still lacking. Recent clinical trials [1–3] failed to show any beneficial effect of early blood pressure lowering on stroke outcome. The large ‘Efficacy of Nitric Oxide in Stroke’ (ENOS) trial—assessing the impact of lowering blood pressure by a ^•^NO-donor [4], using transdermal glyceryl trinitrate—shows a neutral effect on stroke outcome [5]. However, other parameters than the drop in blood pressure are important to be considered. For example, when patients received glyceryl trinitrate very early, within 6 h after symptoms onset, some benefits appear [5, 6]. Therefore, it seems essential to control timing, duration of administration and choice of the active molecule.

In the large field of ^•^NO-donors, *S*-nitrosothiols, in which the ^•^NO moiety is grafted on a sulfur atom, display several advantages compared to marketed prodrugs of ^•^NO: longer half-lives that allow systemic action (*e*.*g*. 8–45 min), low toxicity and no tolerance phenomenon [7, 8]. Especially, *S-*nitrosoglutathione (GSNO), an endogenous low molecular weight *S*-nitrosothiol formed by nitrosation of reduced glutathione, is involved in the storage and transport of ^•^NO. *S*-nitrosoglutathione exhibits higher stability than ^•^NO, mediates protein *S*-nitrosation and plays an important role in vascular signaling [9]. Therapeutic potential of GSNO has been shown through a systemic [10] vasorelaxant effect associated with a potent inhibitory effect of platelet functions [11, 12]. Concerning its impact following stroke, several studies from Khan *et al*. showed that it had a cerebroprotective effect following ischemia/reperfusion [13, 14] when administered intravenously at non hypotensive doses. Whether it may be protective during the acute phase of stroke has never been evaluated in a thromboembolic model.

Despite longer half-lives than ^•^NO, *S*-nitrosothiols still suffer from a lack of stability. The sulfur-nitrogen bond is easily cleaved by several environmental conditions (metallic cations, enzymes, reductants or other thiols) [15]. Thus, the concentration required to reach and protect the brain is difficult to establish. Moreover, the corresponding doses may induce massive acute hypotension, which in the context of stroke may reduce perfusion in the penumbral area and thus increase the infarct size. Therefore, a suitable formulation is still required to offer protection and sustained release of low amounts of ^•^NO, in order to get closer to physiological production and to reach therapeutic effect [16, 17] without inducing any massive and potentially deleterious drop in blood pressure. *In situ* forming implants allow prolonged drug release. They are liquid drug / polymer solutions (generally a biodegradable poly(*D*,*L*-lactide-*co*-glycolide) copolymer) which solidify at the place of injection through polymer precipitation. The drug is early and progressively released during matrix solidification and polymer erosion [18]. We recently demonstrated that entrapping exogenous *S*-nitrosothiol (*S*-nitroso-*N*-acetylpenicillamine) inside *in situ* formulations prevented the massive fall in blood pressure induced by the administration of the same dose of free S-nitrosothiols [17]. Compared to *in situ* implants, *in situ* microparticles, obtained by emulsification of the drug / polymer solution into an additional external phase, are less viscous, easier to inject and show a reduced burst and better sustained release of the drugs [19, 20].

Based on the above, we hypothesized that *in situ* implants and microparticles are adequate formulations as they would enable a controlled release of GSNO while preventing massive hypotension. They could represent the most beneficial way to administer GSNO in a rat model of thromboembolic stroke induced by autologous administration of blood clots, with neither induced nor forced reperfusion in order to be closer to the clinical situation.

For this purpose, we entrapped GSNO into these formulations and measured *in vitro* release profiles. We measured mean and pulse arterial pressures (MAP and PAP) using telemetry in awaken rats in order to demonstrate an *in vivo* sustained delivery of GSNO. We also evaluated the impact of *in situ* implants and microparticles loaded with GSNO on whole-blood platelet aggregation using Multiplate^®^ analyzer. Finally, we evaluated their therapeutic potential in a rat model of thromboembolic stroke. Our aim was to prove if it is possible to prolong GSNO cardiovascular effects, to improve its profile on whole-blood platelet aggregation and to induce cerebroprotection following thromboembolic stroke after a single administration. These preclinical trials will demonstrate the value of the *in situ* dosage forms, particularly for lasting effects.

## Materials and Methods

### Materials and animals

*S*-nitrosoglutathione purified powder was obtained by the reaction of glutathione to NaNO_2_ in an equimolar ratio as described previously [21]. Formulations were prepared as described previously using poly(*D*,*L*-lactide-*co*-glycolide) polymer solutions in *N*-methyl-2-pyrrolidone (25% m/m) [17]. Isoflurane was purchased from Baxter (France), sodium pentobarbitone from Sanofi-Aventis (Libourne, France) and all products for surgery and animal welfare from a veterinary provider (Centravet, France). Adenosine diphosphate agonist, multiplate test cells and hirudin tubes were purchased from Roche Diagnostics (Meylan, France).

All experiments were performed in accordance with the European Community guidelines (2010/63/EU) for the use of experimental animals and for the respect of the 3 Rs’ requirements for Animal Welfare (I. Lartaud permit n° 54–5; F. Dupuis permit n° 54–105, French Ministry of Agriculture, Paris, France). The protocols and procedures were approved by the advisory regional ethical committee on animal experiments (Comité d’Ethique Lorrain en Matière d’Expérimentation Animale, CELMEA): project “SNOtélém” CELMEA-2012-0006 and project n° 00385.01, February, 13th 2014).

### *In situ* formulation preparation

Formulations were prepared as described previously using poly(*D*,*L*-lactide-*co*-glycolide) polymer solutions in NMP (25% m/m) [17]. GSNO was dissolved in the polymer solution just before use (5% m/m for the dose of 30 mg/kg; 1.25% m/m for the dose of 7.5 mg/kg), without heating. *In situ* implants were obtained by injecting directly the GSNO/polymer/solvent mixture (0.3 g of whole formulation). For *in situ* microparticles, 0.3 g of the GSNO-containing polymer solution was emulsified through a polypropylene connector with 0.3 g of an external phase (sesame oil 96%, aluminium monostearate 2% and Span 80 2% m/m) immediately before injection [17]. Formulations were prepared using sterile bottles and syringes. The two doses evaluated *in vivo* (7.5 and 30 mg/kg) were administered with the same volume of polymer. Exact received doses of GSNO were determined by weighing the syringe/needle system before and after injection.

### *In vitro* release assay

0.3 g of *in situ* implants and 0.6 g of *in situ* microparticles were injected into glass hemolysis tubes (VSM, France) filled with 5 mL of phosphate buffer saline (PBS) 0.148 M pH 7.4 pre-warmed at 37°C. The tubes were incubated in a horizontal shaker at 75 rpm and 37°C (22L WNB and SV 14/22, Memmert, Germany). The release medium was carefully harvested and replaced with 5 mL pre-warmed buffer at each sampling point (1, 4, 7 and 24 h). GSNO concentrations were determined with the colorimetric Griess-Saville and Griess methods according to previously reported protocols [21]. Experiments were run in triplicate.

### Telemetry and injection of formulations

The experiments were conducted on 2 series (n = 7 per series) of 4 to 10 month-old male normotensive outbred Wistar rats (Rj/Han: Wi; Janvier, France). Animals had mild restriction to standard rat chow (A04, Safe, France, 20 g per day) in order to maintain their body weight around 500 g until the end of experiments (10 month-old) and drank water (Aqua-clear^®^, Culligan, USA) *ad libitum*. At 4 months of age, rats were equipped with telemetric devices (PA-C40, Data Sciences International, USA; abdominal aorta) under isoflurane anesthesia and aseptic surgery (48 h analgesia with acetaminophen 60 mg/kg/day *per os*, then 1 month recovery). Each rat was used as its own control. All the injections were performed between 11 and 12 a.m., subcutaneously (21-G needle) under transient isoflurane anesthesia in order to standardize the protocol [17] and to reduce stress due to injections. PBS was used as control in telemetry experiments in order to avoid the washout period needed for the degradation of unloaded polymeric matrix. Our preliminary studies showed that injection of unloaded microparticles (the most complex control) did not change significantly arterial pressures *vs*. PBS (data not shown).

**Fig 1:**
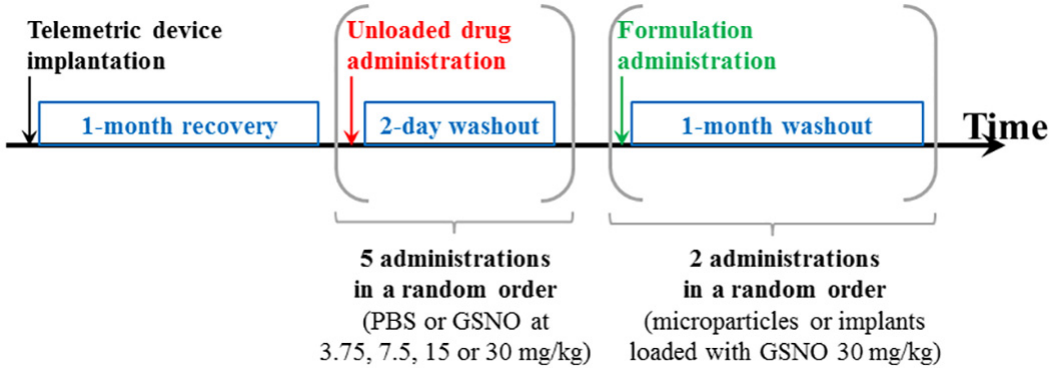
Timeline of the administrations of PBS, free GSNO and formulations loaded with 30 mg/kg GSNO in the first series of Wistar rats. PBS: phosphate buffered saline; GSNO: *S*-nitrosoglutathione.

In the first series, each rat received PBS, then 3.75 to 30 mg/kg (random order) of free GSNO separated by a 2-day wash-out period, then the 2 formulations loaded with GSNO at 30 mg/kg at distinct injection sites (random order, 1-month wash-out period, Fig 1). All those experiments were ended before rats were 10 months old. Basal mean arterial pressure remains stable with age in normotensive Wistar rats [22]. The dose response curves for free GSNO (3.75, 7.5, 15 and 30 mg/kg) were built on the basis of the work of Zanini *et al*. [23].

The doses chosen to be loaded in the formulations were a low and a high dose of GSNO, in order to surround and characterize the best release when measured *in vitro* and *in vivo*. As the lowest free dose (3.75 mg/kg) produced very limited and barely significant variations in blood pressure, we feared it would preclude us to demonstrate an *in vivo* sustained delivery of GSNO. Therefore, we chose 7.5 mg/kg as the lower dose to be included into the formulations.

In order to avoid excessive stress due to repeated experiments conducted on the same animals, a second series of rats was used to receive PBS and the two formulations loaded with GSNO at 7.5 mg/kg following the same protocol (all the administrations made before the rats were 10 months old).

Hemodynamic parameters were recorded (Ponemah 5.1 software, Data Sciences International, USA, 250 Hz sampling rate) in awaken rats. Mean arterial pressure (MAP, area under the pressure wave for each valid cardiac cycle), pulse arterial pressure (PAP, difference between the maximum and minimum pressure values of each pressure wave) and heart rate (HR, number of valid cardiac cycles per min) were recorded during 30 min and averaged on the last 5 min to obtain baseline values.

For each dose of free GSNO, MAP, PAP and HR were recorded continuously for 4 h and their variations *vs*. each corresponding baseline values were averaged on 1 min every 5 min. For GSNO-loaded *in situ* implants and microparticles, MAP, PAP and HR were recorded continuously for 3 days and their variations averaged on 5 min every 15 min. Areas under the curve (pressure AUC) were calculated for each 5 min segment and then averaged for each day (D) or night (N) period. At the end of the experiments, animals were euthanized (pentobarbitone 120 mg/kg *i*.*p*.).

Changes in MAP evaluated the *in vivo* dilating effect of GSNO on arteries and arterioles. Changes in PAP were analyzed as *S*-nitrosothiols are known to induce large venodilation [24]. Venodilation would decrease venous return and cardiac preload and thus decrease stroke volume, the main factor controlling PAP. Therefore, PAP would decrease. The second factor controlling PAP, aortic wall elasticity, does not change following •NO-donors administration [25].

### Analysis of whole-blood platelet aggregation

In order to get closer to physiological conditions and to perform tests with a minimal delay after sampling and with minimal blood volumes, platelet aggregation was assessed using whole-blood impedance aggregometry (Mutliplate^®^ analyzer, Roche Diagnostics) and adenosine diphosphate (ADP) stimulation [26]. Male Wistar rats (n = 58, 5–10 months of age) were divided into 8 groups receiving either PBS, unloaded microparticles, free GSNO at 7.5 or 30 mg/kg, GSNO-loaded microparticles or implants at 7.5 or 30 mg/kg. These doses were chosen on the basis of the telemetry results and to enable direct comparison between acute and prolonged delivery of the same dose of GSNO.

For ethical considerations, the number of blood samples was limited to 5 per animal, based on a previous paper using the same Multiplate^®^ analyzer [27, 28]. Samples were harvested 24 h before and 1, 24, 48, 72, 144, 240 and 312 h after treatments. Thus, more than 5 animals were included in the groups receiving *in situ* formulations (as a prolonged effect was expected) and the sampling was spread all over the 312 hours, in order to cross the results and not have two different populations. Results were analyzed daily and the decision to continue or not the blood sampling on the following days was taken on the basis of physiologically relevant differences observed or not with prior measurements. Several precautions were anticipated to ensure the measurements could still be performed in a blinded way. First, drug administrations were realized in a different room, in a random order and over several days. A first experimenter put the animals (selected on the basis of the daily analysis) into a heated chamber in a random order (5 minutes between each rat). The second experimenter, in charge of blood sampling and aggregometry measurements, only knew in which order the animals had to be removed from the heated chamber and therefore had no possible way to identify the treatments received by the animals. After resting at 37°C for 30 min in order to dilate caudal veins, a small tail incision was made to collect blood (900 μL) into S-Monovette hirudin tubes (Sarstedt, Nümbrecht, Germany). Briefly, 300 μL hirudin blood samples were diluted 1:2 into an isotonic NaCl solution using Mutliplate^®^ test cells and incubated for 3 min at 37°C under stirring conditions. Then, platelet aggregation was triggered with ADP (5 μM final concentration, 5 min after sampling) and recorded for 10 min. Upon activation following agonist addition, platelets adhere and aggregate on test cell's electrodes. This induces a change in impedance directly related to the amount of aggregated platelets. Area under the impedance curve (aggregometry AUC) was used for data analysis as classically considered as the most global and relevant parameter to assess platelet aggregation as a whole. At the end of the experiments, animals were euthanized (pentobarbitone 120 mg/kg *i*.*p*.).

### Thromboembolic stroke

All the surgery, drug administration, scoring and measurements were performed in a blinded way. Thromboembolic stroke was induced in male Wistar rats (CRL:WI(han), Charles River, France) weighing 375–400 g according to Niessen *et al*. [29] with some modifications concerning clot preparation. Briefly, 400 μL of fresh blood were sampled at the tail vein for spontaneous forming of autologous blood clots. The blood was clotted for 110 min, then fragmentation was induced by repeated injections through a sterile needle. Five clot fragments of standardized size (1.5 mm long; 0.3 mm of diameter) were selected and drawn in a 50-cm-long polyethylene (PE30) catheter filled with NaCl 0.9%. The size and number of clot fragments were previously determined to induce reproducible embolism of the middle cerebral artery and allow sufficient sensitivity to detect beneficial effects of pharmacologic agents (data not shown). Clot fragments were injected into the right internal carotid 2 h after the initial blood sampling. Briefly, rats were anesthetized with 2% isoflurane in air. Rectal temperature was maintained at 37.5 ± 0.5°C with the use of a feed-back controlled heating-pad. The right pterygopalatine artery and occipital branches of the right external carotid artery were ligated. Five minutes before clots injection, the tubing containing clots was inserted in the right external carotid artery up to the bifurcation with the internal carotid artery. The clots were injected along with 350 μl of NaCl 0.9%. Both left and right common carotids were temporally occluded during clots injection. The surgical procedure was completed within 20 min.

Two hours after clot embolism, the animals were classified into 5 categories in a blinded manner: A no symptoms, B light symptoms (no replacement of the left paws after manipulation), C moderate symptoms (circling behavior or paretic limbs detected without manipulation), D severe symptoms (inability to walk) and E unconscious rat. This rough estimate of the severity of the immediate symptoms was not influenced by post-anesthesia as we previously observed that sham-operated rats were fully awaken 2 hours after surgery and were all classified in the A category. Rats from the A and E categories were excluded from the study as they were likely to show no detectable lesion (A) or to die within 12 hours (E, previously unpublished data). Included rats were then allocated to the 4 groups of treatment by using a stratified randomization method to balance severity of the immediate symptoms between groups. Immediately after scoring and group allocation, *i*.*e*. 2 h after stroke, the rat received either unloaded microparticles (n = 11), free GSNO at 7.5 mg/kg (n = 11) or *in situ* forming implants (n = 10) or microparticles (n = 9) loaded with GSNO 30 mg/kg (see below for sample size determination). These doses were chosen on the basis of the aggregometry and blood pressure results. As only the formulations loaded with the highest dose of GSNO significantly modified the aggregation profile, we did not include formulations loaded with 7.5 mg/kg in this experiment. For ethical reason, it was not possible to test free GSNO at 30 mg/kg as the acute hypotension would probably have killed the animals. We used the dose of 7.5 mg/kg as the free control.

### Neurological score calculation

An extended neurological examination consisting in a succession of 15 sensori-motor items was performed in a blinded way 24 and 48 h after embolism. A total neurological deficit score was calculated for each rat by adding the 15 items sub scores (maximum 160). Each item was scored as follows: 0, no impairment on left side; 5, moderate impairment; 10, severe impairment. For spontaneous walking, a specific scale was used (0, no impairment; 5, circling when pulled by the tail; 10, circling; 20, unable to walk). The 15 items (for a more detailed description of the items, see De Ryck *et al*. [30], Bederson *et al*. [31] and Nedelmann *et al*. [32]) are: reaction to visual stimuli approaching from left, whisker movements on left side, left whisker sensitivity (reaction to stimulation of whisker), left ear hearing (normal or no reaction to acoustic stimuli), spontaneous walking (see above), left forelimb flexion (extension of the forelimbs when the rat is pulled by the tail with forepaw in contact with the floor), inability to fully extend left hind limb (extension of the hind limbs when the rat is pulled by the tail with forepaws in contact with the floor), torso twisting (rotation of the body 3 times consecutively when lifted by the tail), visual forelimb placing (coordinated grasping when lifted by the tail toward a beam), left forelimb replacement (paw replacement when the forelimb is pushed backward), left hind limb replacement (paw replacement when the hind limb is pushed backward), proprioceptive hind limbs retrieval (the retrieval of limb is scored as the rat is pushed backward toward the edge of a table until the limb suddenly fall), tactile forward placing of forelimbs (placing of the limbs when the dorsal aspect of the paw are pushed against the table, the rat’s head maintained 45°C upward to avoid visual and tactile (*i*.*e*. whisker) contacts with the table), forepaw digits sensitivity (reaction to slight pinch of each digit), lips sensitivity (reaction to slight pinch of the upper lip). Except for walking and torso twisting, normal reaction on the right side (ipsilateral to the infarct) was verified before scoring the left side (contralateral to the infarct).

### Edema and infarct size evaluation

After the last neurological examination (48 h post stroke), rats were euthanized with pentobarbitone (120 mg/kg *i*.*p*.) and a 2,3,5-triphenyltetrazolium chloride (TTC) staining was performed to determine volume of edema and infarcted brain volume corrected for edema [33]. The brain was quickly removed and cooled down in ice-cold saline for 5 min. Brain was cut in 2-mm thick coronal slices using a brain matrix (Zivic Instruments, Pittsburgh, PA, USA). The sections were stained for 20 min in a 2% TTC solution at 37°C. For quantification, the slices were digitalized with an Epson Perfection V37 scanner (Seiko Epson Corporation, Suwa, Japan). Volume of edema and infarcted brain volume corrected for edema were calculated from area measurements on each picture slides using ImageJ software (NIH, Bethesda, MD, USA).

### Statistical power and sample size calculation for the stroke experiments

Given the acknowledged variability of the thromboembolic stroke model, we performed an *a priori* determination of sample size and power analysis using G*Power 3 software [34] to ensure robust and reliable results.

Data mixed from several unpublished standardized pharmacological studies (same exclusion criterion as explained above) indicate that the injection of 5 clot fragments in control rats (n = 38) induces a neurological score at 48 h of 54 ± 35 (mean ± standard deviation), whereas rats treated with the fibrinolytic agent Alteplase (n = 32) present a score of 32 ± 28. Moreover, we conducted a preliminary study to verify the effects of empty microparticles or free GSNO (3.75 and 7.5 mg/kg) 2 h after clots administration to approximate their impact on neurological score at 48 h. We found that neither empty microparticles nor free GSNO induced a reduction in neurological score (n = 5 per group, data not shown).

Based on these results, we formulated the following *a priori* hypothesis on mean neurological scores at 48 h for the 4 groups (k = 4) planned in the present study: 55 for PBS and free GSNO (7.5 mg/kg) treated rats. We hypothesized an optimistic score of 15 for GSNO-loaded (30 mg/kg) *in situ* microparticles and implants treated rats. The latter assumption is based on the work of Khan *et al*. (on a cerebral ischemia/reperfusion model) who observed a reduction of -66% of neurological score following a single administration of a low dose of GSNO given intravenously at the time of reperfusion [35]. As we expected our formulations to induce a continuous release of GSNO while inducing only mild hypotensive effect, we assumed their impact on neurological score would be at least as important as the results obtained by Khan *et al*. The overall standard deviation was estimated at σ = 30 units of neurologic score (*i*.*e*. a standard deviation of 55% for control animals). Given these hypotheses, for a statistical power of > 80% (*i*.*e*. type-2 error probability β < 20%) and a type-1 error probability α < 5%, the ideal calculated sample size was: n = 8 per group (actual power = 85%). Based on our previous experiments, we anticipated a survival rate of 70% at 48 h and thus assigned 9 to 11 rats per treatment (depending on the exclusion criterion explained above) to achieve n = 8 animals completing the whole study in each group (animals dying before 48 h being excluded from the analysis).

### Statistical analysis

Results are expressed as means ± s.e.m. Significant differences in blood pressures and HR between PBS and free GSNO were determined by a two-way ANOVA (variables: “time” and “dose”) followed by a post-hoc Bonferroni test. Significant differences between PBS and GSNO-loaded into implants or microparticles were determined in each series (7.5 and 30 mg/kg) by a one-way ANOVA performed on pressure AUC, followed by a post-hoc Bonferroni test. The duration of effect was evaluated as the last time point, which still showed significant difference from the corresponding PBS effect.

Significant differences in platelet aggregation over time were also evaluated within each group by a one-way ANOVA followed by a post-hoc Bonferroni test.

As discrete values, significant differences in neurological score between groups were evaluated by a Kruskal-Wallis analysis followed by a post-hoc Mann-Whitney test for non-parametric values. For more clarity, we used the same statistical test for infarct size and edema volume, even if it is weaker than parametric tests..

The null hypothesis was rejected at p < 0.05. Statistical analyses were performed using GraphPad Prism version 5 (GraphPad Software).

## Results

### *In vitro* release profile

**Fig 2:**
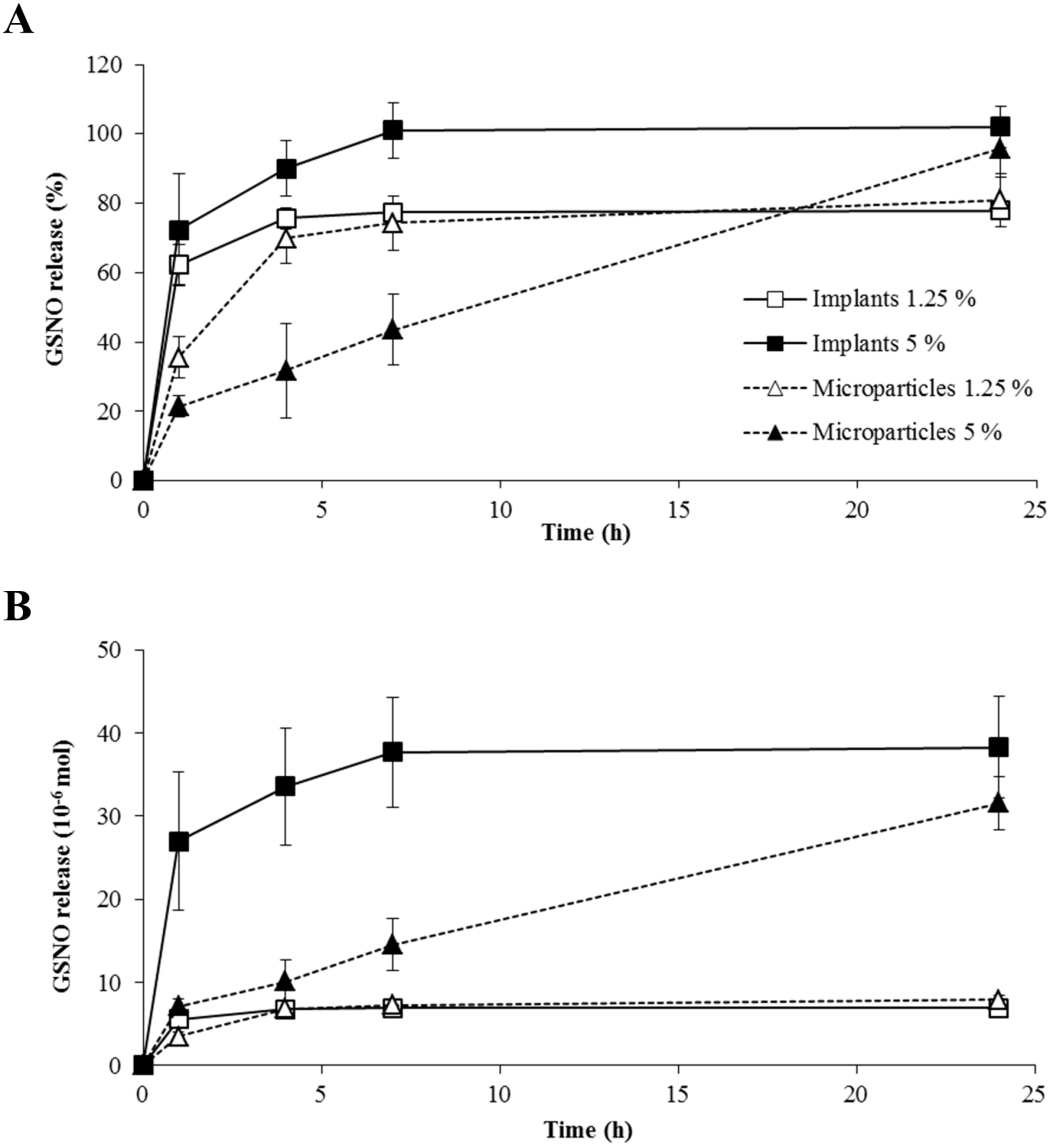
*In vitro* releases of GSNO from *in situ* forming implants and microparticles. GSNO loadings were 1.25% or 5% m/m (corresponding respectively to 7.5 mg/kg and 30 mg/kg of GSNO for *in vivo* experiments) based on the amount of polymer/GSNO/solvent solution. Panel A represents the release of GSNO expressed as % of the initial load; Panel B represents the release of GSNO in absolute values. Values are mean ± s.e.m. (n = 3).

The release profiles of GSNO from *in situ* implants and microparticles are presented in Fig 2. A rapid and important release of GSNO was observed from implants or microparticles loaded with 1.25% m/m of GSNO (corresponding to the dose of 7.5 mg GSNO/kg of body weight, when administered to animals): 75% or more of the loaded quantity of GSNO released before 4 h (Fig 1A). The release of GSNO from implants loaded with 5% GSNO m/m (corresponding to the dose of 30 mg/kg) followed the same pattern. Microparticles loaded with 5% GSNO m/m was the only formulation with a longer phase and a linear release of GSNO from 1 to 24 h (Fig 2A and 2B), suggesting that microparticles loaded with 5% GSNO m/m, rather than implants (even with the same initial load) or microparticles loaded with 1.25%, would be the most interesting formulation to produce a controlled release of GSNO.

### Effects of GSNO-loaded formulations on mean and pulse arterial blood pressures

**Fig 3:**
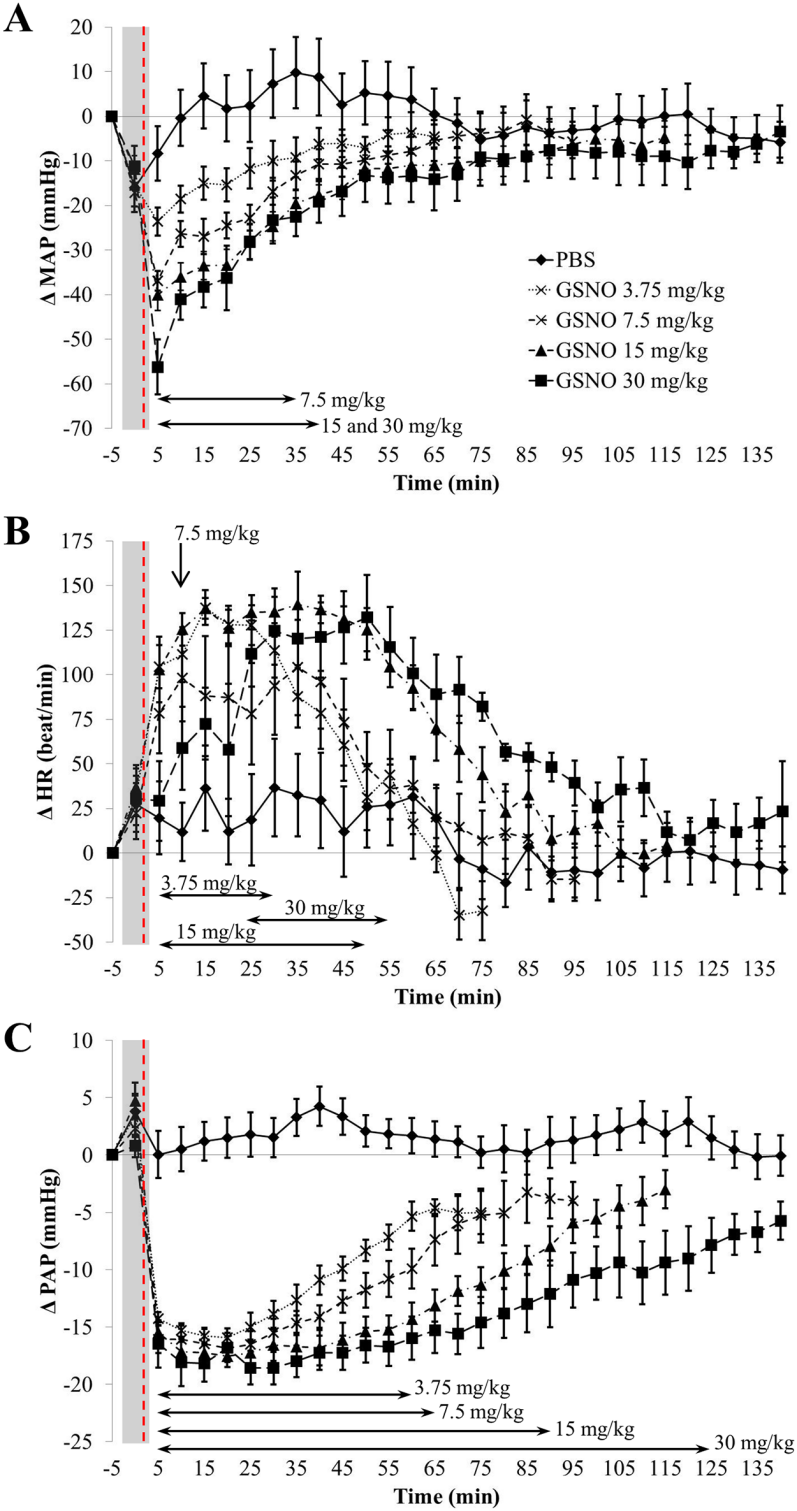
Impact of free GSNO on MAP, (panel A), HR, (panel B) and PAP (panel C). p_interaction_, p_dose_ and p_time_ < 10^−4^ for all parameters (two-way ANOVA; post-hoc Bonferroni test). Each animal (n = 7) received all the treatments. Arrows indicate for each dose the time span where significant differences *vs*. PBS were observed. Grey boxes represent the duration of anesthesia and red vertical dashed lines the time of injection.

Firstly, rats submitted to PBS showed slight increases in MAP and HR (+ 10% *vs*. baseline), during the first 30 to 50 min of waking up following transient anesthesia, which was masked when rats received GSNO (Fig 3).

Free GSNO induced a dose-dependent fall in MAP (-24 ± 3 to -56 ± 6 mmHg at 3.75 to 30 mg/kg) *vs*. baseline values. This fall in MAP lasted 35–40 min (7.5 to 30 mg/kg, Fig 3A) and was associated with a same duration of tachycardia (Fig 3B). PAP fell (– 17 mmHg, *i*.*e*.– 50% *vs*. baseline) from the first 3.75 mg/kg dose. The duration of the fall in PAP was dose-dependent and lasted longer than the decrease in MAP and increase in HR (from 60 min at 3.75 mg/kg to 125 min at 30 mg/kg, Fig 3C).

**Fig 4:**
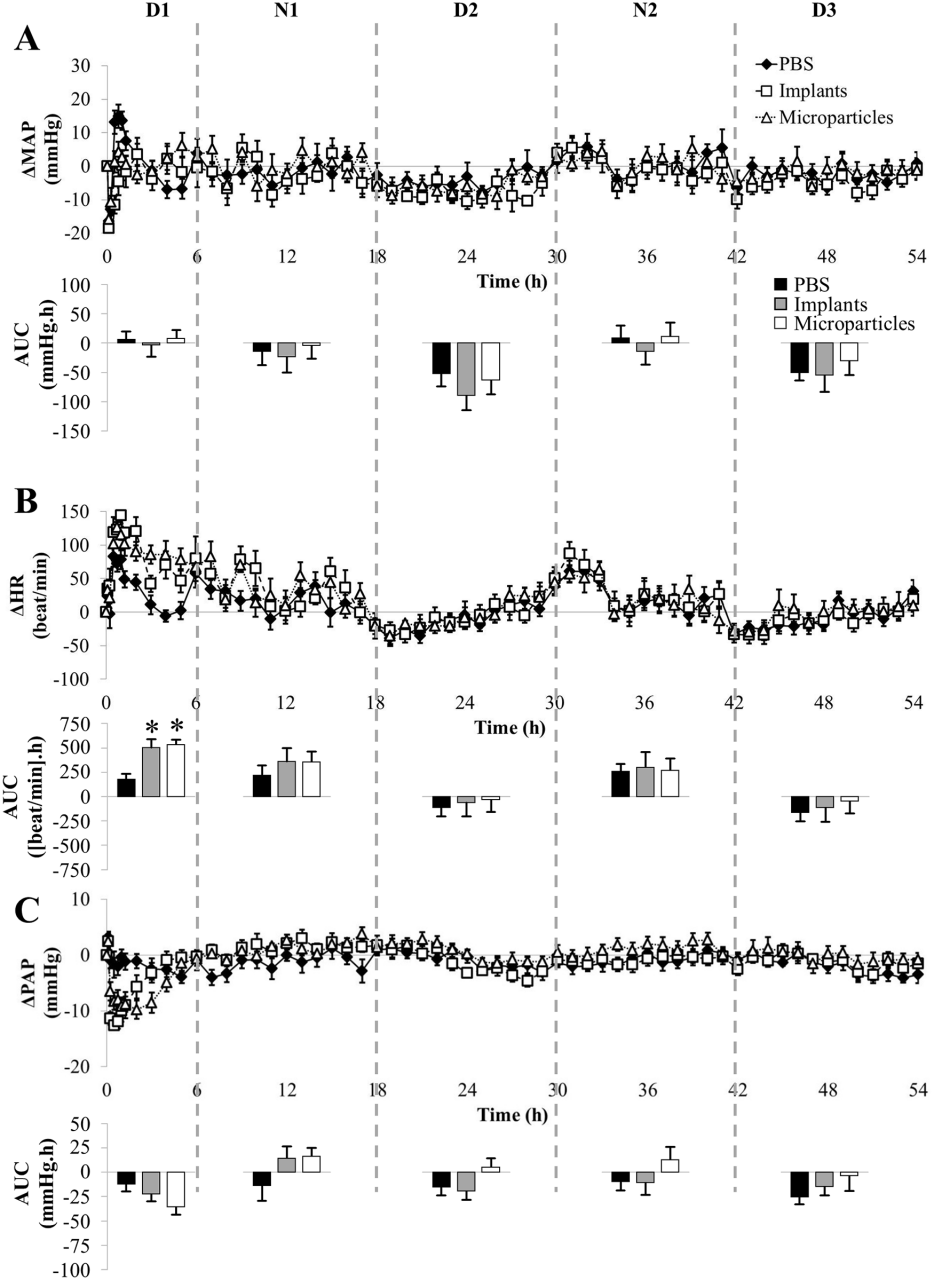
Impact of formulated GSNO at 7.5 mg/kg on MAP (panel A), HR (panel B) and PAP (panel C). Each animal (n = 7) received all the treatments (Implants: 7.4 ± 0.2; Microparticles: 7.7 ± 0.2 mg/kg). Day-night cycles are marked in days (D1, D2 and D3) and nights (N1, N2) periods. For more clarity, after the first hour, only one point for each hour was represented. *: p < 0.05 *vs*. PBS (one-way ANOVA; post-hoc Bonferroni test).

**Fig 5:**
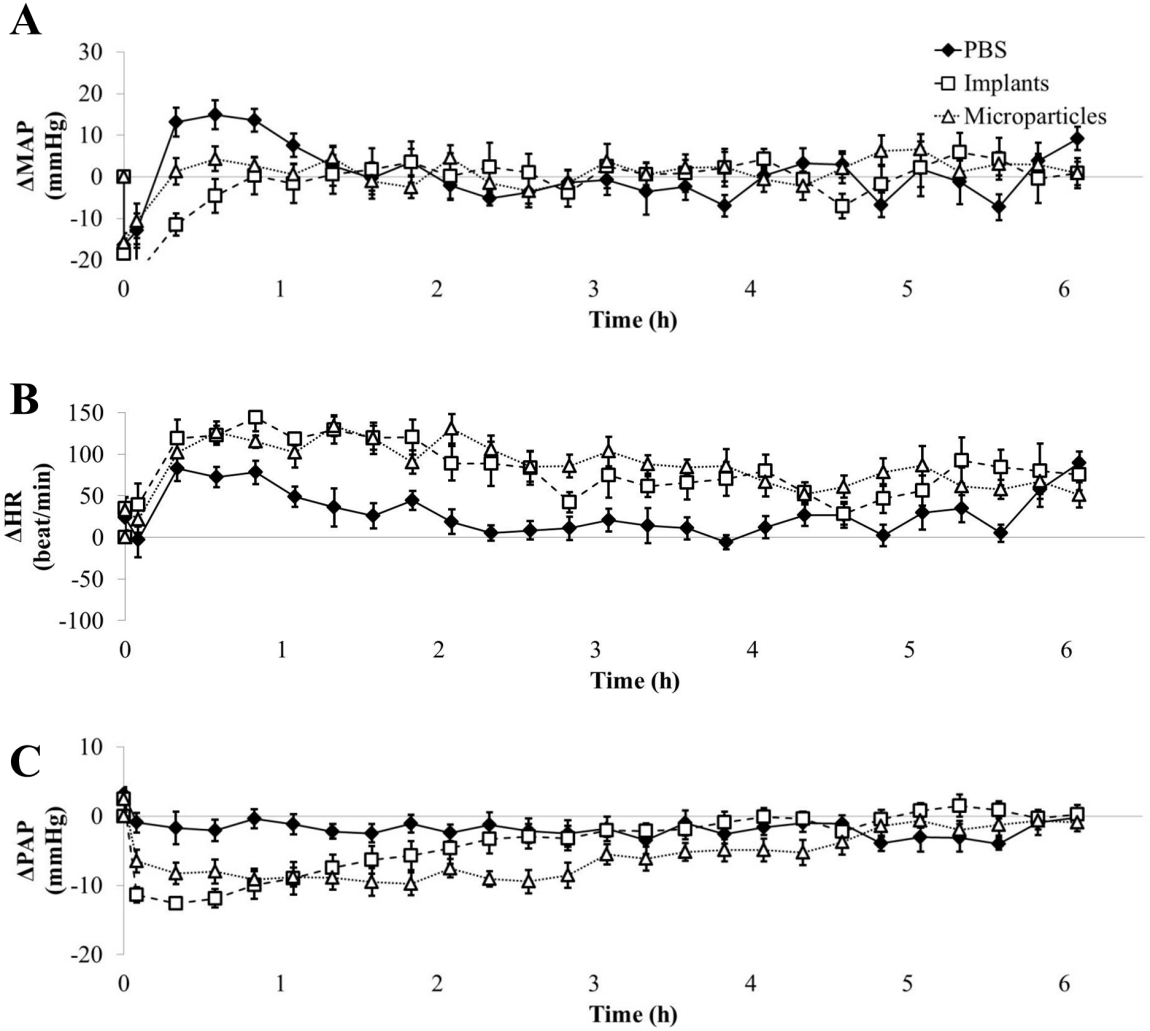
Detailed impact of formulated GSNO at 7.5 mg/kg on MAP (panel A), HR (panel B) and PAP (panel C) within the first half day following administration. Each animal (n = 7) received all the treatments (Implants: 7.4 ± 0.2; Microparticles: 7.7 ± 0.2 mg/kg).

Secondly, when arterial pressure was recorded during 3 days, pressure AUC showed positive values in rats treated with PBS at night in relation with their night activity and negative ones during the day (Fig 4A, AUC). This was not the case for the first half-day (Fig 5), where the positive value may be attributed to strong cardiovascular activity during waking up following anesthesia. GSNO-loaded formulations at the dose of 7.5 mg/kg did not induce hypotension as shown by the AUC values (Fig 4A). However, this lack of significant impact was associated with a significant increase in HR during the first half-day (Fig 4B). Both formulations also induced a transient decrease in PAP (-10 mmHg) that lasted 3 h with implants and 5 h with microparticles (Fig 4C).

**Fig 6:**
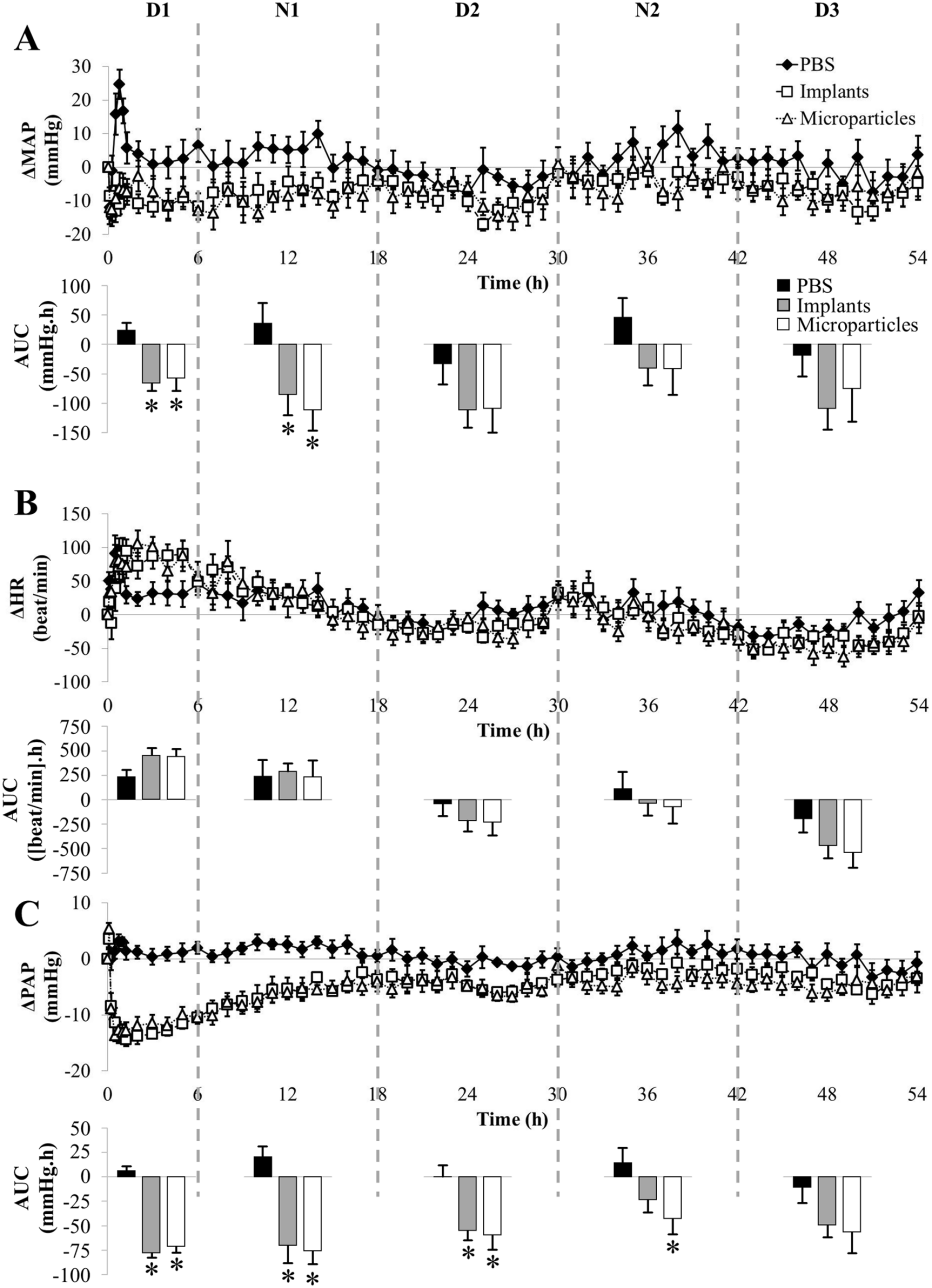
Impact of formulated GSNO at 30 mg/kg on MAP (panel A), HR (panel B) and PAP (panel C). Each animal (n = 7) received all the treatments (Implants: 31.9 ± 0.5; Microparticles 30.9 ± 0.6 mg/kg). Day-night cycles are marked in days (D1, D2 and D3) and nights (N1, N2) periods. For more clarity, after the first hour, only one point for each hour was represented. *: p < 0.05 *vs*. PBS (one-way ANOVA; post-hoc Bonferroni test).

**Fig 7:**
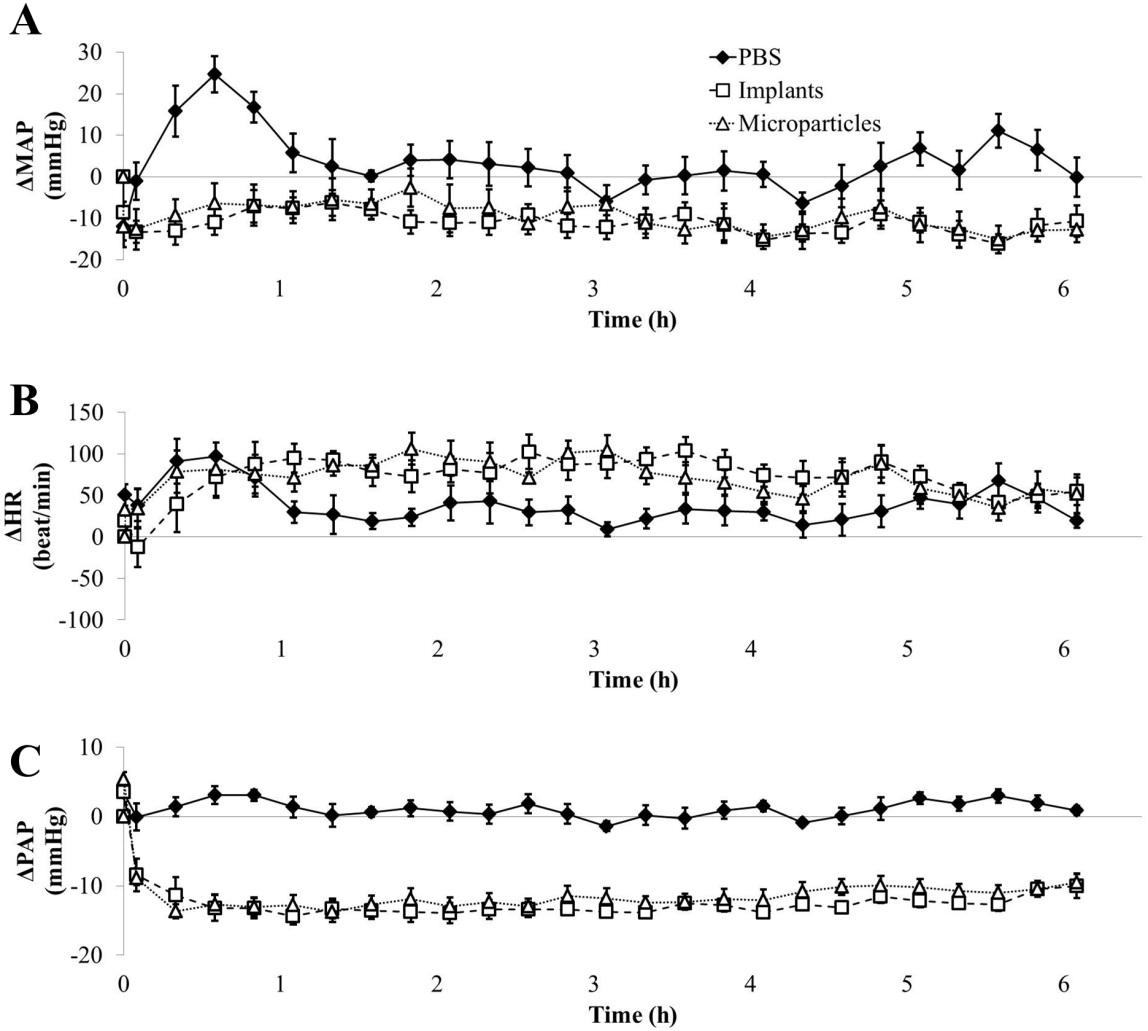
Detailed impact of formulated GSNO at 30 mg/kg on MAP (panel A), HR (panel B) and PAP (panel C) within the first half day following administration. Each animal (n = 7) received all the treatments (Implants: 31.9 ± 0.5; Microparticles 30.9 ± 0.6 mg/kg).

Thirdly, at the dose of 30 mg/kg, GSNO-loaded *in situ* implants and microparticles induced a slight (- 10 mmHg which corresponds to—15% *vs*. baseline) but prolonged hypotension as shown by the significant decrease in MAP AUC values during the first 18 h (Figs 6A and 7), *i*.*e*. a 20-fold longer effect than the corresponding dose of free GSNO.

Both formulations loaded with GSNO at 30 mg/kg induced a slight but non-significant increase in HR during the first half-day (Figs 6B and 7). PAP was reduced to the same extent as free GSNO (- 50% *vs*. baseline, Fig 6C). This decrease in PAP was longer and reached significance till 30 h with *in situ* implants and till 42 h with *in situ* microparticles (Fig 6C), *i*.*e*. a 15 (implants) to 21 (microparticles) fold longer decrease in PAP than the corresponding dose of free GSNO.

### Effects of GSNO-loaded formulations on whole-blood platelet aggregation

**Fig 8:**
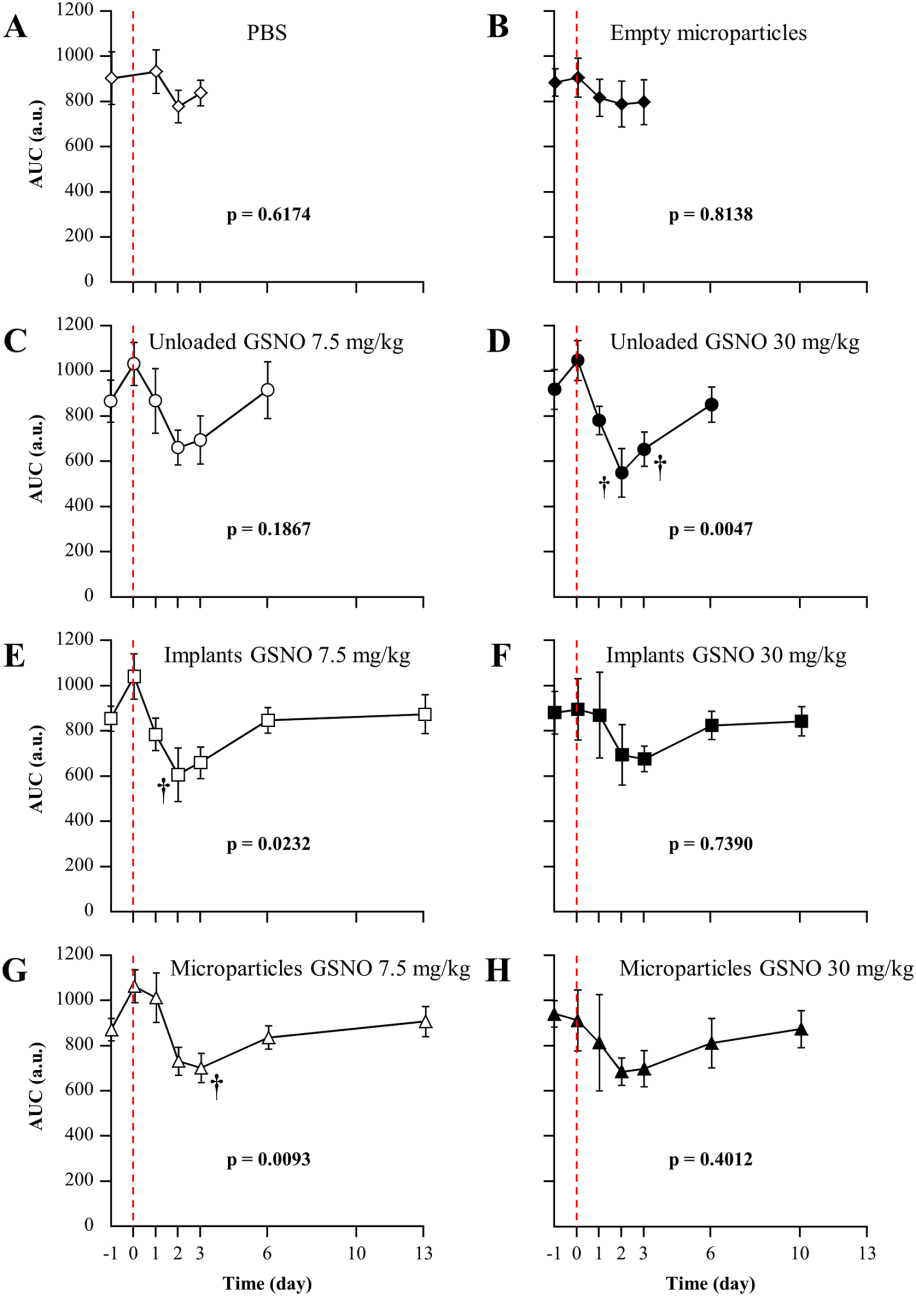
Impact of the treatments on whole blood platelet aggregation (aggregometry AUC). Treatments were: PBS (n = 6), empty microparticles (n = 8), free GSNO at 7.5mg/k g(n = 5) or 30 mg/kg (n = 5), *in situ* implants loaded with 7.5 ± 0.8 mg/kg (n = 7) or 32.1 ± 4.3 mg/kg (n = 10) of GSNO and *in situ* microparticles loaded with 7.9 ± 0.6 mg/kg (n = 7) or 31.4 ± 3.6 mg/kg (n = 10) of GSNO. The red vertical dashed lines represent the time of injection. †: p < 0.05 *vs*. 1 h following substance administration within the same group (one-way ANOVA; post-hoc Bonferroni test).

The effect of GSNO-loaded formulation was tested on whole-blood aggregation. Fig 8A and 8B showed that administration of PBS or unloaded microparticles as well as the repeated blood sampling have minor impacts on platelet functions.

Free GSNO (7.5 and 30 mg/kg, Fig 8C and 8D), as *in situ* implants and microparticles loaded with GSNO at 7.5 mg/kg, showed a biphasic profile (Fig 8E and 8G). An immediate but transient (< 24 h) increase in aggregometry AUC illustrates platelet hyper-aggregability. This phenomenon was followed by an inhibition of platelet aggregation between 48 and 72 h following GSNO administration. *In situ* implants and microparticles loaded with GSNO at 30 mg/kg showed a smoother profile (Fig 8F and 8H, ANOVA along time p > 0.05). Platelet function recovered in all groups before the 6^th^ day.

### Effects of GSNO-loaded formulations following stroke

The survival rates at 48 h were not different amongst groups: 73% in animals receiving empty microparticles, 73% in rats receiving free GSNO at 7.5 mg/kg (n = 8), 67% in rats receiving microparticles loaded with 30 mg/kg of GSNO (n = 6) and 80% in rats receiving implants loaded with 30 mg/kg of GSNO (n = 8).

**Fig 9:**
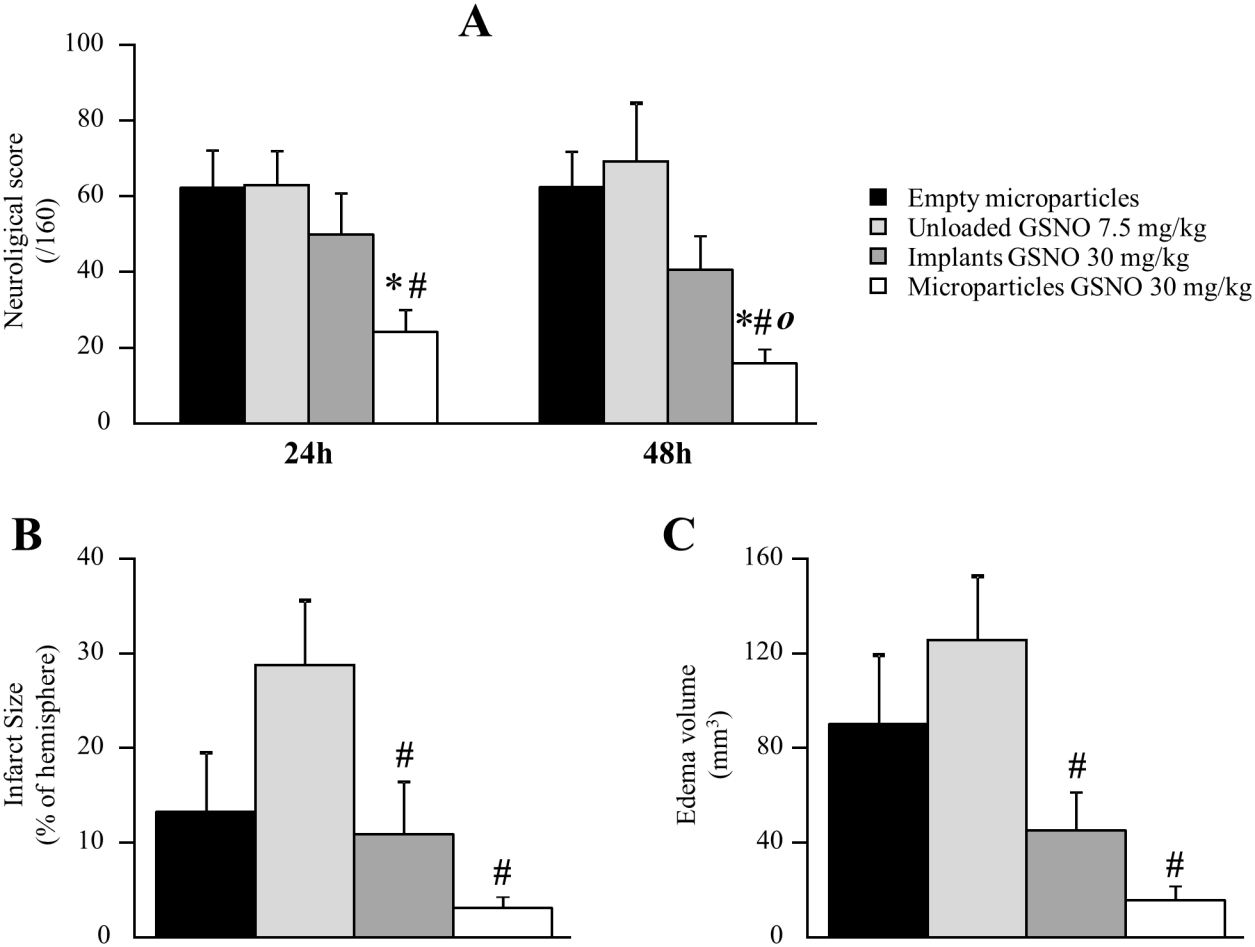
Impact of the treatments on neurological score, infarct size and edema volume. Treatments were: empty microparticles (n = 8), free GSNO at 7.5 mg/kg (n = 8) and *in situ* implants loaded at 30.1 ± 1.5 mg/kg (n = 8) or microparticles at 31.4 ± 0.9 mg/kg (n = 6) of GSNO, injected in male Wistar rats 2 h after thromboembolic stroke induction. Neurological score (panel A, maximal score 160) was measured 24 and 48 h after stroke induction, infarct size (panel B) and edema volume (panel C) 48 h after. *: p < 0.05 *vs*. empty microparticles; #: p < 0.05 *vs*. free GSNO at 7.5 mg/kg; *ο*: p < 0.05 *vs*. *in situ* implants (Kruskal-Wallis; post-hoc Mann-Whitney test).

Free GSNO at 7.5 mg/kg, injected in rats 2 h after stroke induction, did not reduce neurological score measured 24 and 48 h after (Fig 9A), nor infarct size or edema volume compared to control rats injected with empty microparticles (Fig 9B and 9C).

*In situ* GSNO-loaded microparticles at 30 mg/kg, but not *in situ* implants, reduced neurological scores by 62–75% compared to empty microparticles or free GSNO at 24 and 48 h (Fig 9A). At 48 h, the effect of GSNO-loaded microparticles on neurological score was significantly different from GSNO-loaded implants.

At 48 h, GSNO-loaded microparticles divided infarct size by 10 and 8 compared to free GSNO. *In situ* implants only reduced infarct size and edema volume by 2.5 to 3 fold *vs*. free GSNO (Fig 9B and 9C), but the differences between *in situ* implants and microparticles did not reach statistical significance.

## Discussion

In the present study, *in situ* implants and microparticles loaded with 30 mg/kg of GSNO strongly attenuated the amplitude and extended the duration of the effects of GSNO on MAP and PAP compared to free GSNO. This sustained effect lasted up to 42 h for microparticles, *i*.*e*. 20-fold longer than when induced by the same dose of free GSNO. *In situ* formulations also flattened the platelet aggregation profile and protected the brain against the neurological consequences of thrombi-induced stroke.

Since their development by Bodmeier *et al*. in the early 2000s [36], several reports have been made about the optimization of the *in situ* forming microparticles. The *in vitro* release profiles obtained in the present report confirmed a more linear and controlled release of GSNO with microparticles rather than implants. Microparticles are formed more slowly than implants, as the external oily phase slows down the exchange between the solvent and body fluids. Furthermore, the burst (initial release during the first hours) is higher for implants as the solvent/body fluids exchange is fast, and part of the GSNO might be driven out of the formulation with the solvent [18, 20]. The superiority of the *in situ* microparticles in terms of injectability and drug release profile (sustained release with limited burst) has been proven by several teams [17, 19, 37].

Our data also showed that these linear profiles depend on the payload of GSNO, as microparticles loaded with the lowest dose produced a faster and less linear release than those with the highest dose of GSNO. This difference is probably related to a threshold in solubility. In the highest dose loaded microparticles, GSNO could be under a non-dissolved form (suspension) and therefore an additional dissolution step would be required before its release, modulating the profile.

Altogether, these results suggest that *in situ* microparticles loaded with GSNO at 30 mg/kg would be the most interesting formulation to achieve a controlled release of GSNO *in vivo*. However, *in situ* formulation behavior remains highly dependent on the *in vitro* or *in vivo* environment. The differences in physical constraints and rates of the water/solvent exchange highly interfere with the three main phases of *in situ* formulations, *i*.*e*. formation/precipitation of the matrix, drug release and scaffold degradation. As a result, correlations between *in vitro* and *in vivo* drug release profiles are often challenging [17, 18, 38]. Moreover, in our experiments, it is difficult to parallel with pharmacokinetics, as the literature classically describes numerous pre-analytical and analytical difficulties to monitor plasmatic concentrations of GSNO [39], in relation with its poor stability (*e*.*g*. light, temperature and enzymes) [40]. Therefore, physiological concentration of GSNO is still a matter of debate, ranging from 0.00135 ± 0.00046 to 1.78 ± 0.76 μM [39]. While we failed to evaluate pharmacokinetics of the supplementation of GSNO, our *in vivo* pharmacodynamics data show dose-dependent effects.

*In situ* GSNO-loaded 30 mg/kg implants and microparticles strongly prolonged the hemodynamic effects of GSNO. To the best of our knowledge, only one other formulation of GSNO has been tested in animals. After vaginal administration, GSNO-loaded films multiplied by 10 the duration of the local vascular effect of GSNO (3.5 h *vs*. 20 min for the unformulated drug) [41]. When loaded at 30 mg/kg, formulations led to an attenuated and slight hypotension (around—10 mmHg instead of—56 mmHg) which lasted 20 fold longer than when obtained with the same dose of free GSNO. When loaded with GSNO at 7.5 mg/kg, the prolongation of the effect on MAP was barely 2 fold in duration, suggesting, in accordance with the profile of the *in vitro* kinetic, different profiles for releasing GSNO depending on the doses charged into the formulations.

Subcutaneous injection of GSNO, loaded or not into formulations, also led to a decrease in PAP, which lasted longer for *in situ* microparticles (42 h) than implants (30 h). This decrease in PAP is most likely related to a decrease in stroke volume in relation with venodilation, as other factors regulating stroke volume may be ruled out: (i) the improvement of cardiac function due to hypotension-induced decrease in cardiac afterload or to baroreflex-stimulated sympathetic response is not high enough to compensate the fall in stroke volume, (ii) a direct positive myocardial effect of *S*-nitrosothiols does not seem to occur in our experiments, otherwise this would increase stroke volume [42].

The Multiplate^®^ analyzer allows to test platelet functions in the presence of red blood cells, which is of particular importance given the interaction of ^•^NO and hemoglobin [43]. Interestingly, while it is commonly admitted that ^•^NO and ^•^NO-donors inhibit platelet functions [11, 44], our results show that free GSNO displays a biphasic impact on whole-blood platelet aggregation. The trend to immediate but transient platelet hyper-aggregability was rapidly followed by a more pronounced and prolonged hypo-aggregability. Such a biphasic response has been recently documented [45]. The authors showed either activation or inhibition of platelets soluble guanylyl cyclase (sGC), depending on ^•^NO concentration.

Both GSNO *in situ* formulations at 7.5 mg/kg show a similar biphasic profile, suggesting that formulations loaded with low doses do not allow a significant controlled and prolonged release of GSNO *in vivo*. However, when loaded at 30 mg/kg, *in situ* microparticles and implants flattened the aggregation profile with no transient hyper-aggregability and an inhibitory effect that failed to reach statistical significance. This also supports the idea of a low grade and prolonged GSNO release from high dose loaded *in situ* formulations which agrees with the profiles obtained by *in vitro* kinetic of release. The lower aggregability certainly participated in the better protection against thromboembolic stroke observed with GSNO-loaded formulations.

Free GSNO at 7.5 mg/kg did not improve neurological score (neither at 24 h nor at 48 h) and tended to increase infarct size (Fig 9B, p = 0.103 *vs*. unloaded microparticles). We agree that the small sample size in our experiments could hamper the interpretation of this trend, but these results seem in discrepancy with the protective effect of GSNO reported by Khan *et al*. [13, 14, 35]. This could be explained by a dual effect of GSNO at 7.5 mg/kg after ischemic stroke. First, GSNO has been reported to produce a direct protective effect on the neurovascular unit by reducing inflammation, oxidative stress and peroxynitrite formation [14, 35]. However, at 7.5 mg/kg, free GSNO produces a transient but strong hypotension (Fig 3), potentially leading to the increase in infarct size by reducing perfusion in penumbral tissue. Furthermore, in our stroke model using blood clots, the transient hyper-aggregability induced by free GSNO may also contribute to enlarge infarct size by extending the size of the clots. These negative effects of free GSNO could not be detected in Khan *et al*.’s experiments as they used a nylon monofilament model (not blood clots) and GSNO intravenously at a non-hypotensive dose (1 mg/kg). Hence, the larger infarct size in the free GSNO group measured in our experiments may be related to the acute hypotensive effect of GSNO while the direct beneficial effects of GSNO were preventing the rise in neurological score.

GSNO had a beneficial effect on the consequences of stroke when administered formulated into microparticles or implants at 30 mg/kg. *In situ* microparticles significantly reduced neurological score, even more than implants (as well as infarct size and edema volume, but without reaching statistical significance).

By their capacities to induce slight but sustained release of GSNO, the formulations contributed to these beneficial effects. Our formulations prevented the negative impacts of GSNO on aggregation and blood pressure. The controlled delivery of GSNO by the *in situ* formulations produces only a slight decrease in MAP (-10 mmHg, which is smaller than the reduction of blood pressure targeted in clinical trials assessing blood pressure control in the acute phase of stroke [1–3]) while avoiding an acute deleterious drop in pressure. The long duration of this slight hypotension highlights the sustained release of GSNO. As the clinical trials [1–3] have shown a neutral effect of early blood pressure reduction on stroke outcome, we can hypothesize that the hypotension induced by our formulation was neither deleterious, nor beneficial. In our previous sets of experiments (see Methods), unformulated GSNO at the low dose of 3.75 mg/kg (inducing a transient decrease in MAP of similar degree to that induced by GSNO-loaded microparticles at the dose of 30 mg/kg), did not improve nor worsen the outcome after stroke. In the present study, we did not measure blood pressure in stroked animals. We cannot rule out that the hypotension produced by our formulations could be potentially more deleterious in case of preexisting hypertension, as hypertension can increase sensitivity to blood pressure lowering [46]. In such context, formulations with a lower dose (e.g. 15 mg/kg) could be tested as it could be a good compromise between less (or shorter in time) hypotensive and direct neuroprotective effects.

Beside blood pressure, *in situ* formulations loaded with GSNO at 30 mg/kg also attenuated platelet hyper-aggregability. This also probably contributes to avoid the negative effect of free GSNO and thus to decrease the consequences of thromboembolic stroke. Furthermore, this suggests that these formulations could be safe and efficient in case of intracerebral hemorrhage. GNSO has been shown to be beneficial in case of subarachnoid hemorrhage in rats [47]. The longer reduction in PAP could also contribute to the efficacy of the formulations, as high PAP is associated with poor outcome following stroke [48].

While GSNO-loaded *in situ* microparticles (and probably better than implants) appear as very promising formulations for ^•^NO sustained effect in thromboembolic stroke, we have to remain cautious as we did not observe any significant impact on mortality following stroke. Our treatments were able to improve the outcome of the animals surviving stroke, but did not improve the survival rate. Moreover, the long term impact of the formulations has not been evaluated in this study, animals being sacrificed 48 h after stroke. The actual gap between the drug release time and the life-span of the polymeric matrix [19] should not be a problem in the context of stroke, as these formulations could be used as a ‘single shot’ acute emergency treatment producing a sustained effect.

## Conclusion

By measuring arterial pressure parameters in rats, we presently obtained the proof of concept of a sustained and prolonged GSNO effect using *in situ* implants and *in situ* microparticles, based on degradable poly(lactide-*co*-glycolide). Microparticles loaded with GSNO at 30 mg/kg showed the best profile with longest effects on PAP, witnessing a sustained release. Compared to smaller doses of free GSNO, both 30 mg/kg-loaded implants and microparticles attenuated the GSNO-induced hypotensive effect (MAP), flattened the platelet aggregation profile and decreased infarct size and edema volume following stroke. Loaded microparticles improved neurological score. Therefore, GSNO-loaded *in situ* formulations could be useful in case of cerebral ischemic diseases.

## Supporting Information

S1 Archive Images of the brains obtained from the rats receiving empty microparticles. Coronal slices were stained using TTC.
(ZIP)

S2 Archive Images of the brains obtained from the rats receiving free GSNO at 7.5 mg/kg. Coronal slices were stained using TTC.
(ZIP)

S3 Archive Images of the brains obtained from the rats receiving implants loaded with GSNO 30 mg/kg. Coronal slices were stained using TTC.
(ZIP)

S4 Archive Images of the brains obtained from the rats receiving microparticles loaded with GSNO 30 mg/kg. Coronal slices were stained using TTC.
(ZIP)

S1 Table Raw data of the stroke experiment. (XLSX)
