# Supplementary figures and images for: In Situ Microparticles Loaded with S-Nitrosoglutathione Protect from Stroke
Source: PLoS One. 2015 Dec 8;10(12):e0144659. doi: 10.1371/journal.pone.0144659 (PMC4672927; doi:10.1371/journal.pone.0144659)

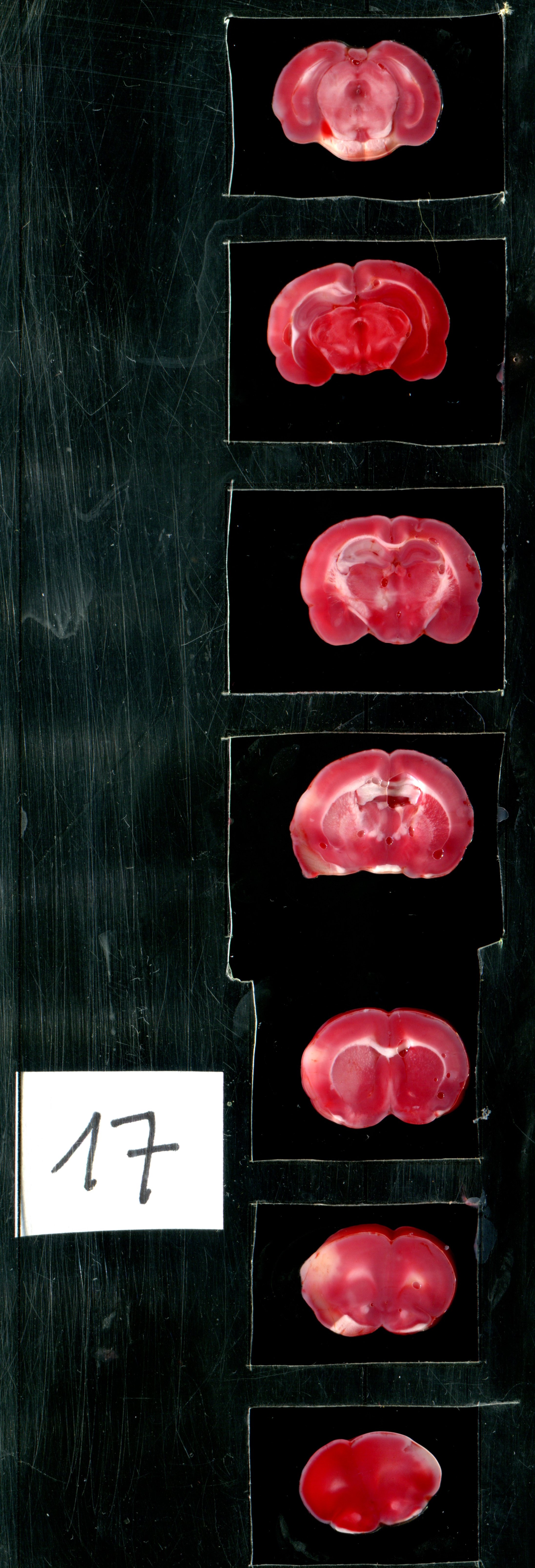

Supplement: S1 Archive — Coronal slices were stained using TTC. (ZIP) [file pone.0144659.s001.zip › G0401_GSNO_R117bis.jpg]

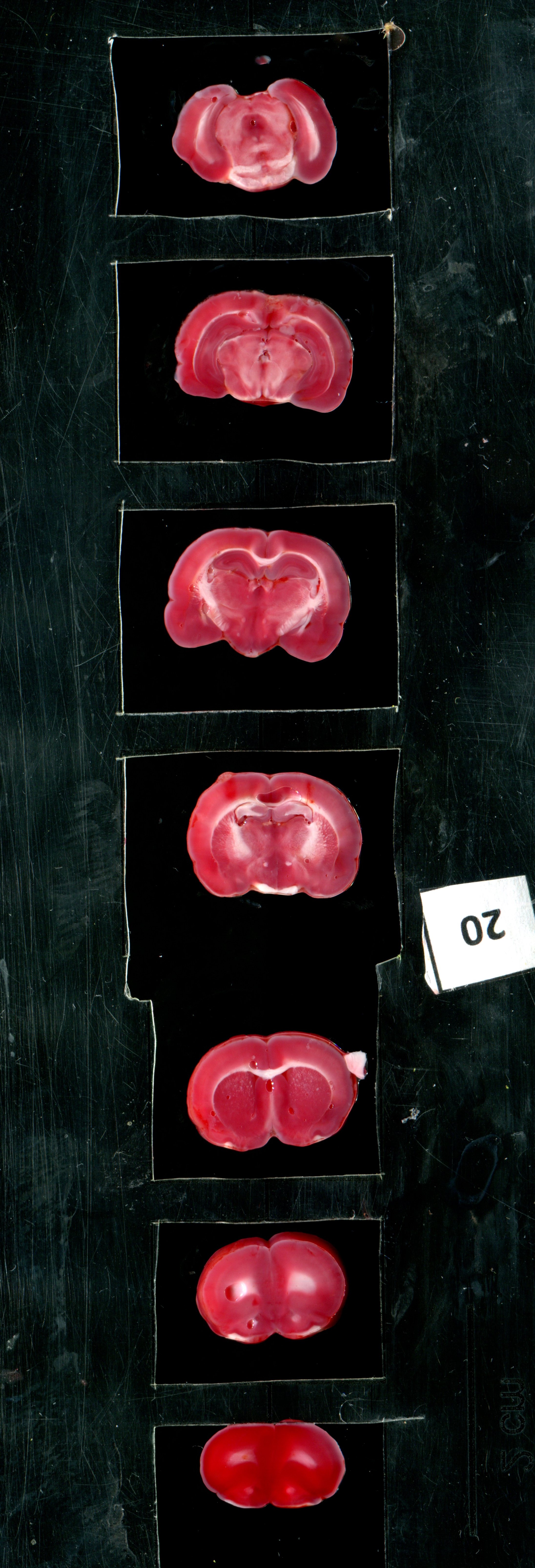

Supplement: S1 Archive — Coronal slices were stained using TTC. (ZIP) [file pone.0144659.s001.zip › G0401_GSNO_R120bis.jpg]

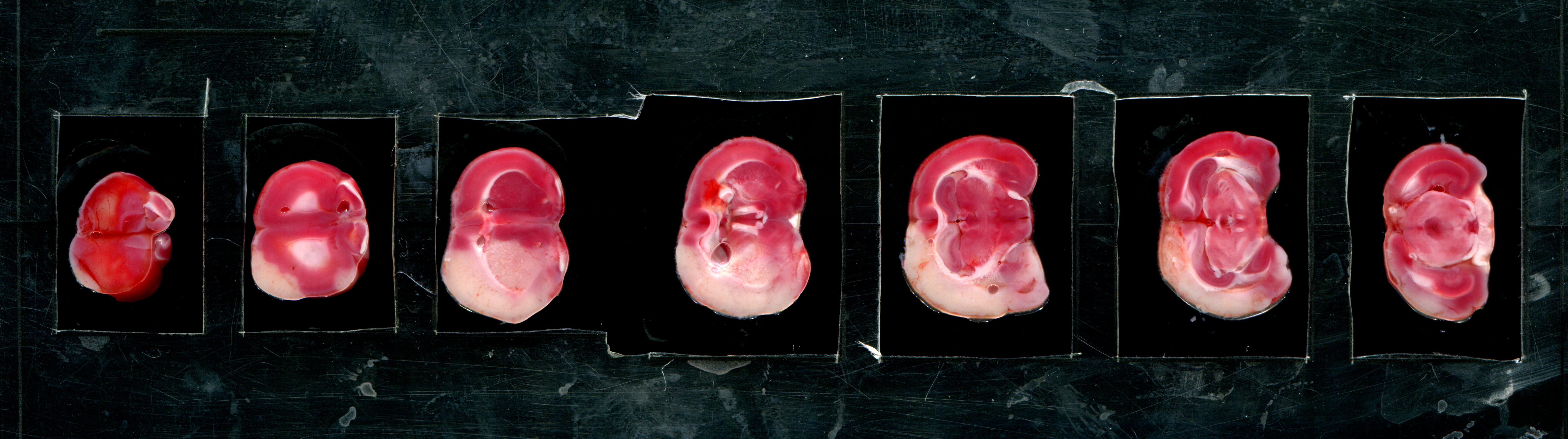

Supplement: S1 Archive — Coronal slices were stained using TTC. (ZIP) [file pone.0144659.s001.zip › G0401-2_GSNO_R11bis.jpg]

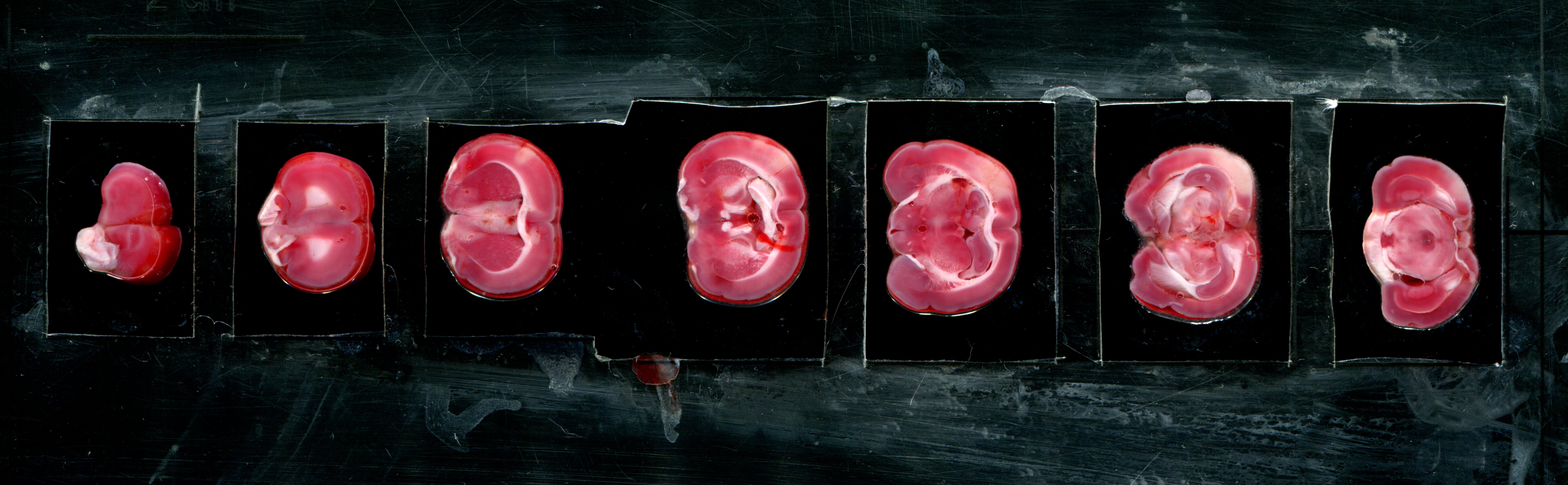

Supplement: S1 Archive — Coronal slices were stained using TTC. (ZIP) [file pone.0144659.s001.zip › G0401-2_GSNO_R43bis.jpg]

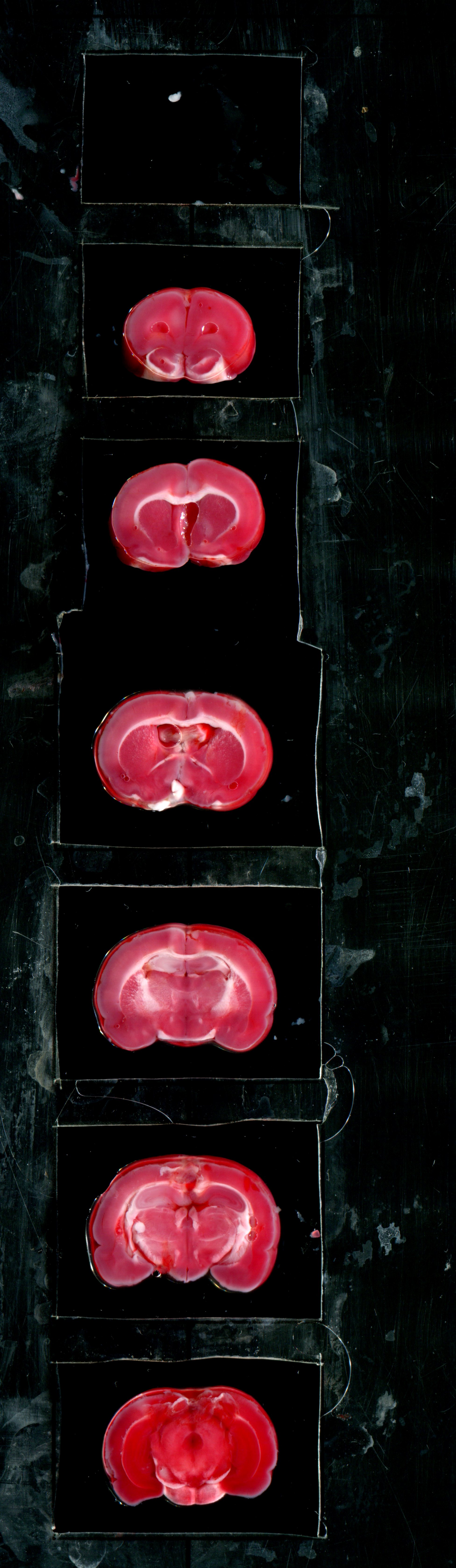

Supplement: S1 Archive — Coronal slices were stained using TTC. (ZIP) [file pone.0144659.s001.zip › G0401-2_GSNO_R50bis.jpg]

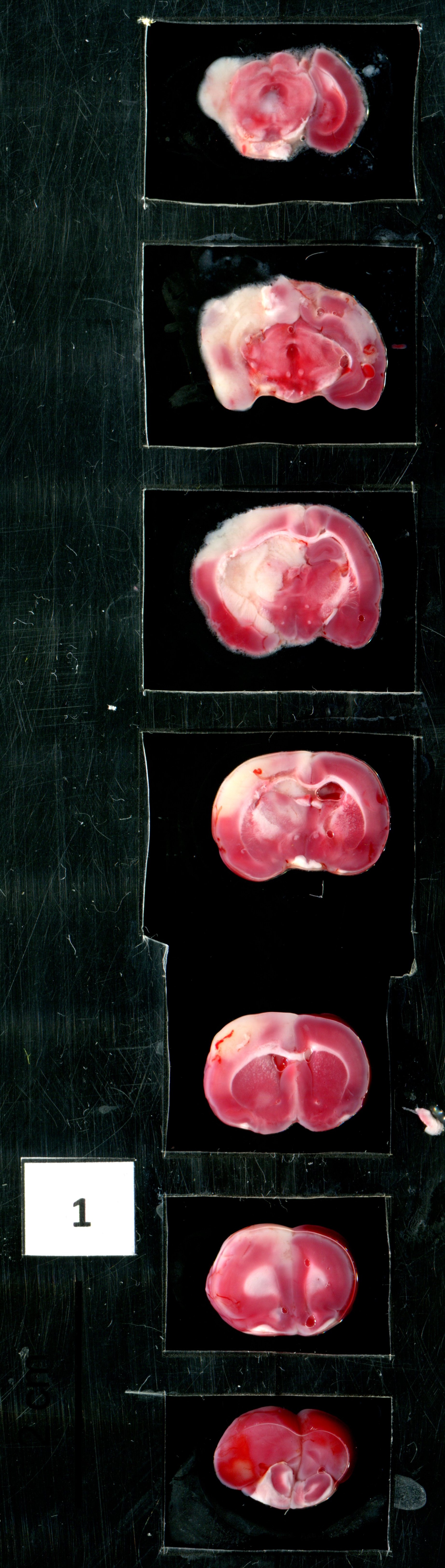

Supplement: S1 Archive — Coronal slices were stained using TTC. (ZIP) [file pone.0144659.s001.zip › G0401_GSNO_R101bis.jpg]

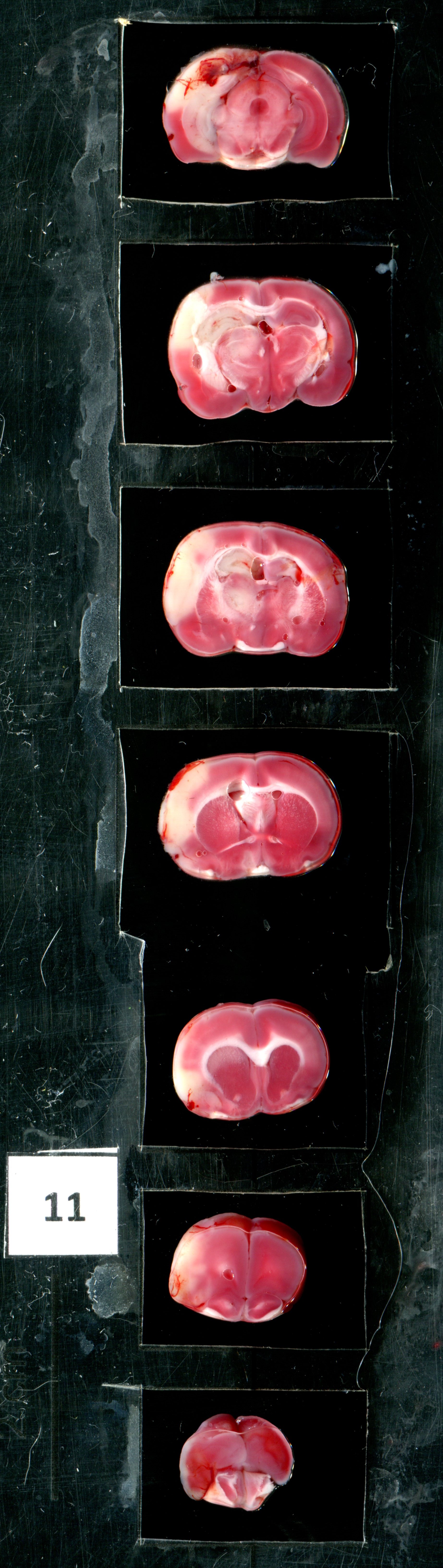

Supplement: S1 Archive — Coronal slices were stained using TTC. (ZIP) [file pone.0144659.s001.zip › G0401_GSNO_R111bis.jpg]

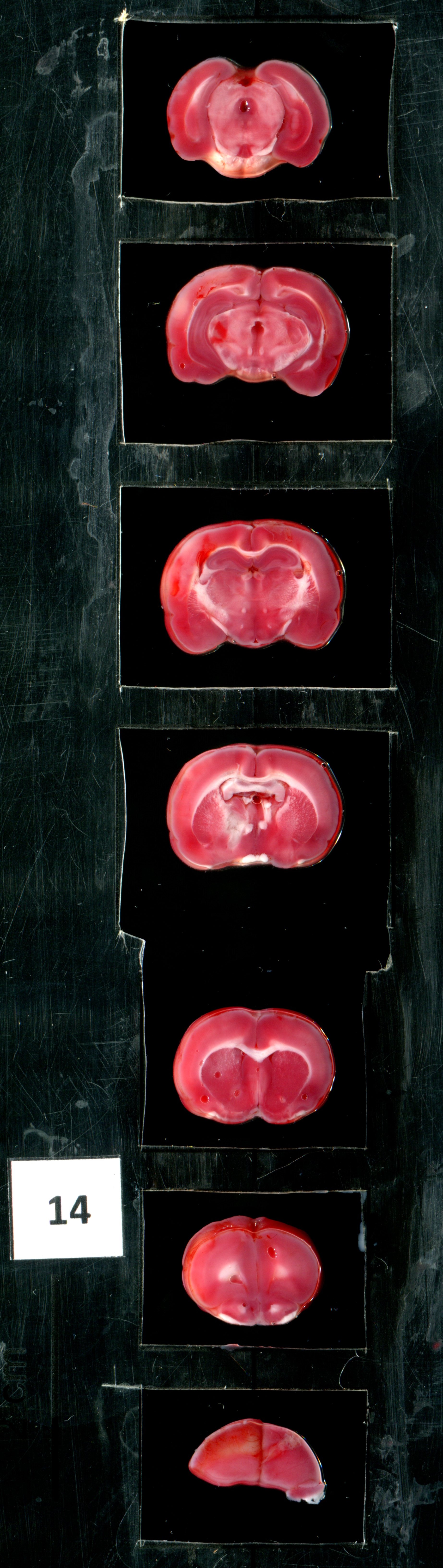

Supplement: S1 Archive — Coronal slices were stained using TTC. (ZIP) [file pone.0144659.s001.zip › G0401_GSNO_R114bis.jpg]

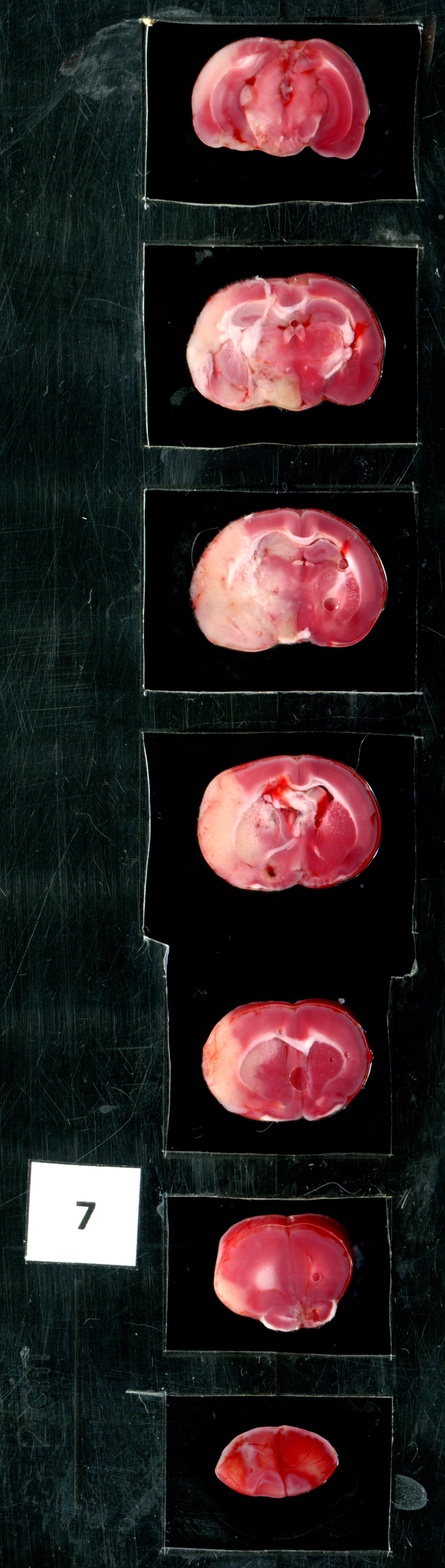

Supplement: S2 Archive — Coronal slices were stained using TTC. (ZIP) [file pone.0144659.s002.zip › G0401_GSNO_R107bis.jpg]

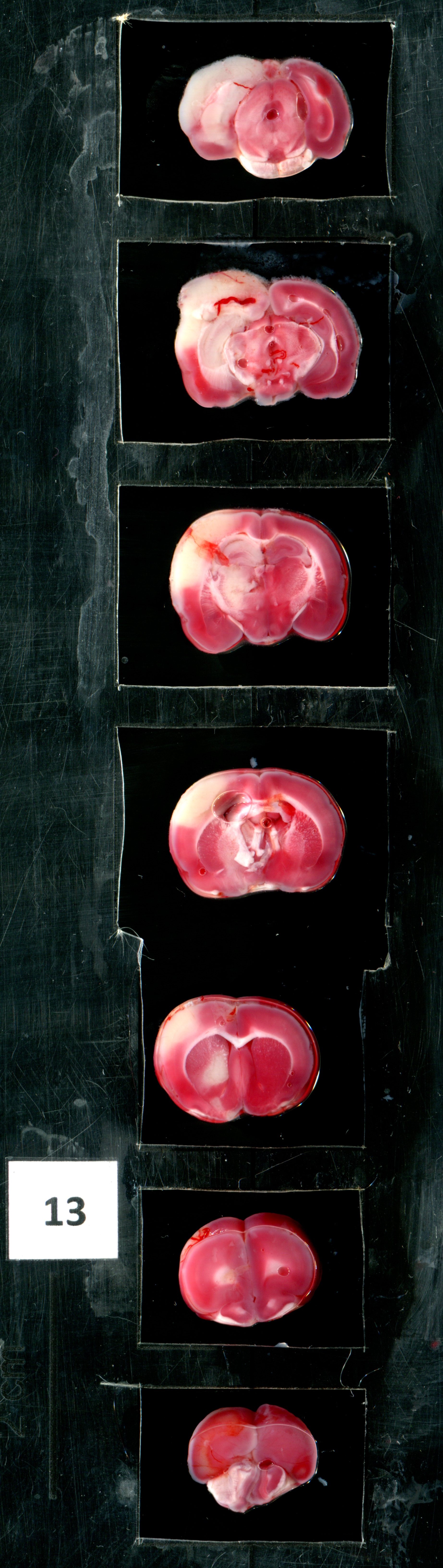

Supplement: S2 Archive — Coronal slices were stained using TTC. (ZIP) [file pone.0144659.s002.zip › G0401_GSNO_R113bis.jpg]

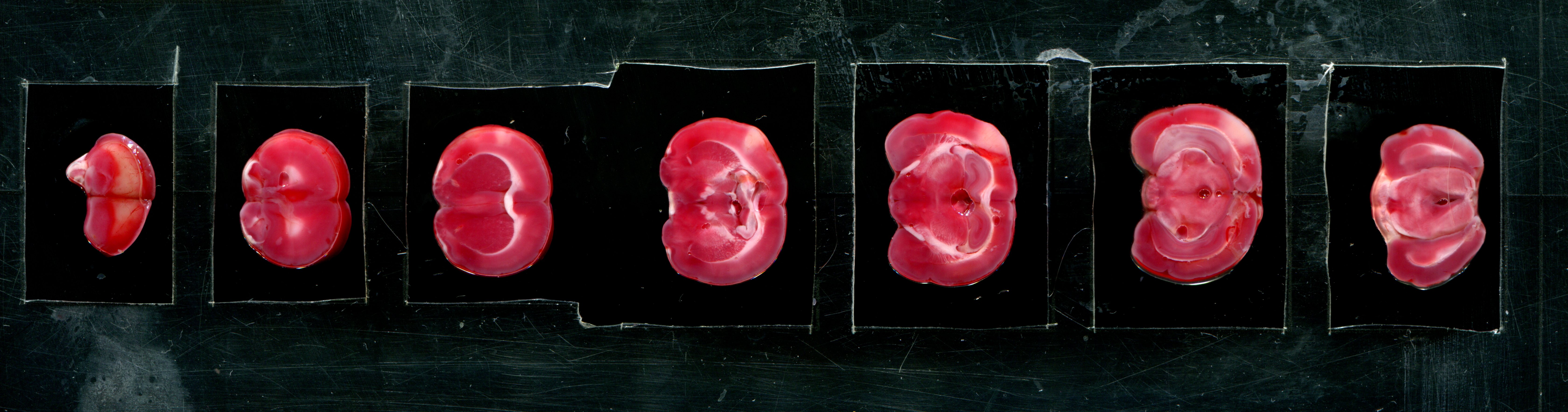

Supplement: S2 Archive — Coronal slices were stained using TTC. (ZIP) [file pone.0144659.s002.zip › G0401-2_GSNO_R2bis.jpg]

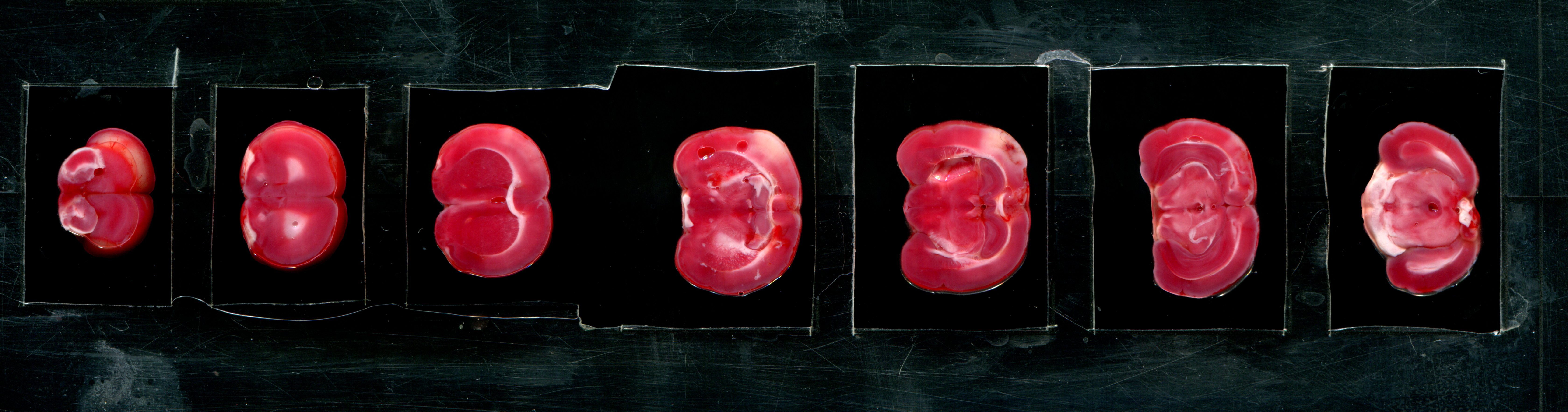

Supplement: S2 Archive — Coronal slices were stained using TTC. (ZIP) [file pone.0144659.s002.zip › G0401-2_GSNO_R5bis.jpg]

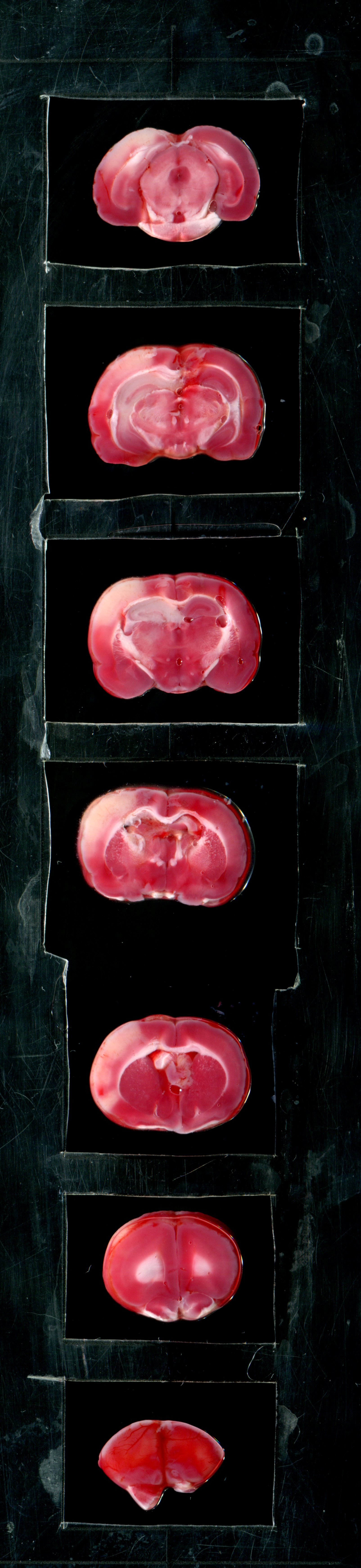

Supplement: S2 Archive — Coronal slices were stained using TTC. (ZIP) [file pone.0144659.s002.zip › G0401-2_GSNO_R38bis.jpg]

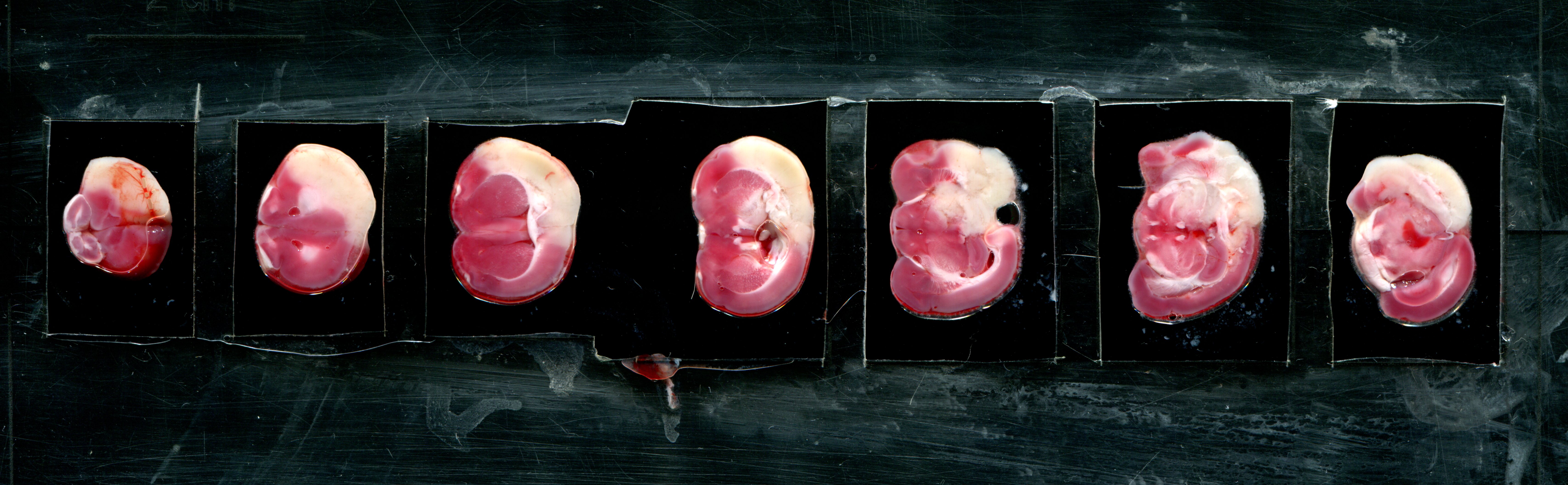

Supplement: S2 Archive — Coronal slices were stained using TTC. (ZIP) [file pone.0144659.s002.zip › G0401-2_GSNO_R42bis.jpg]

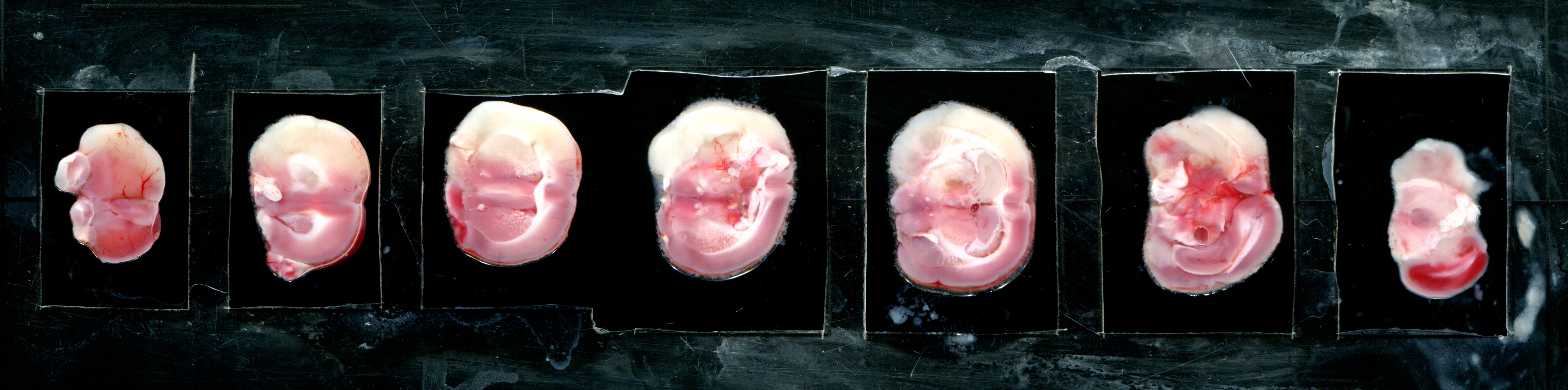

Supplement: S2 Archive — Coronal slices were stained using TTC. (ZIP) [file pone.0144659.s002.zip › G0401-2_GSNO_R46bis.jpg]

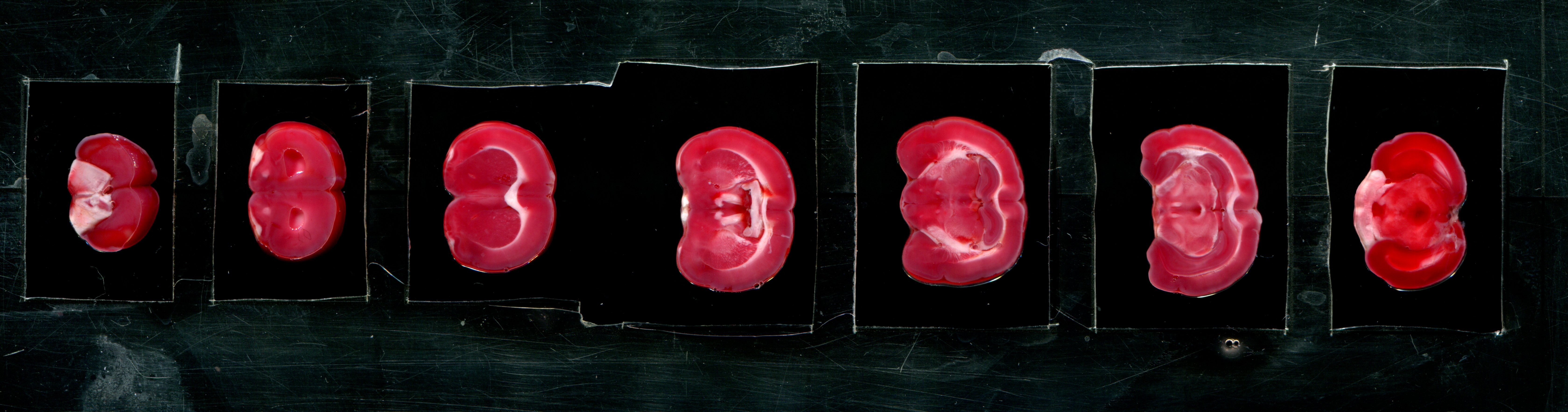

Supplement: S3 Archive — Coronal slices were stained using TTC. (ZIP) [file pone.0144659.s003.zip › G0401-2_GSNO_R4bis.jpg]

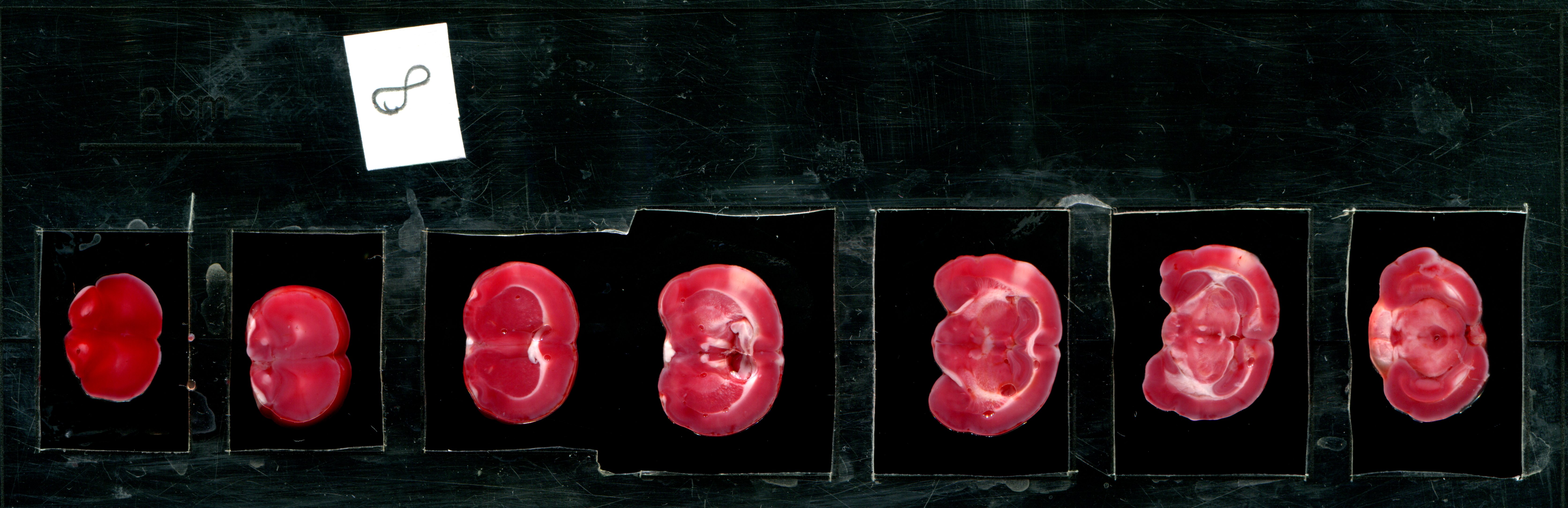

Supplement: S3 Archive — Coronal slices were stained using TTC. (ZIP) [file pone.0144659.s003.zip › G0401-2_GSNO_R8_bis.jpg]

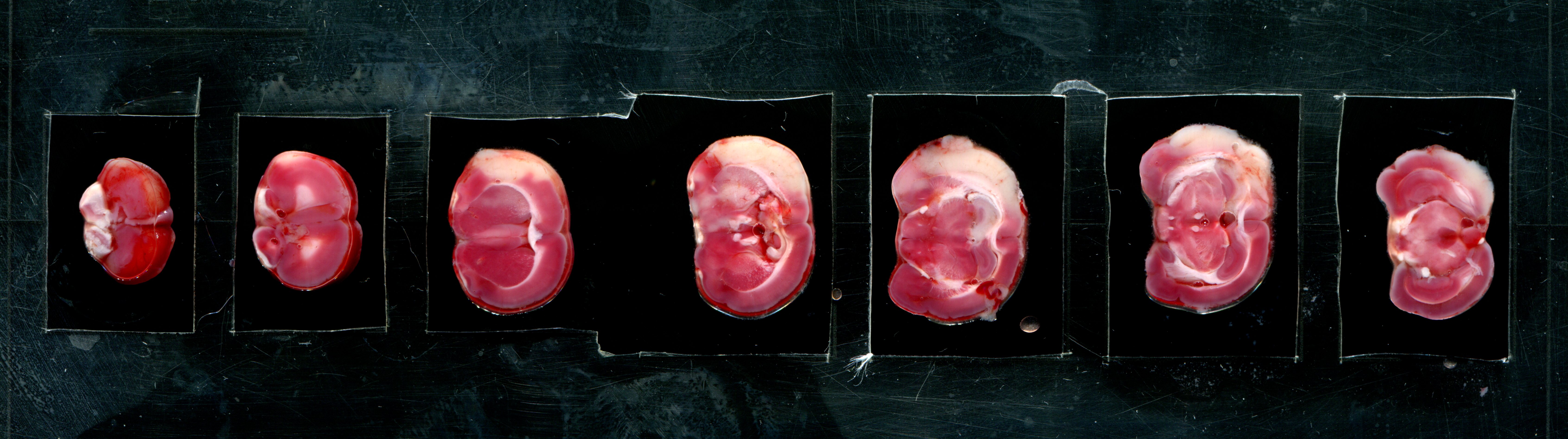

Supplement: S3 Archive — Coronal slices were stained using TTC. (ZIP) [file pone.0144659.s003.zip › G0401-2_GSNO_R9bis.jpg]

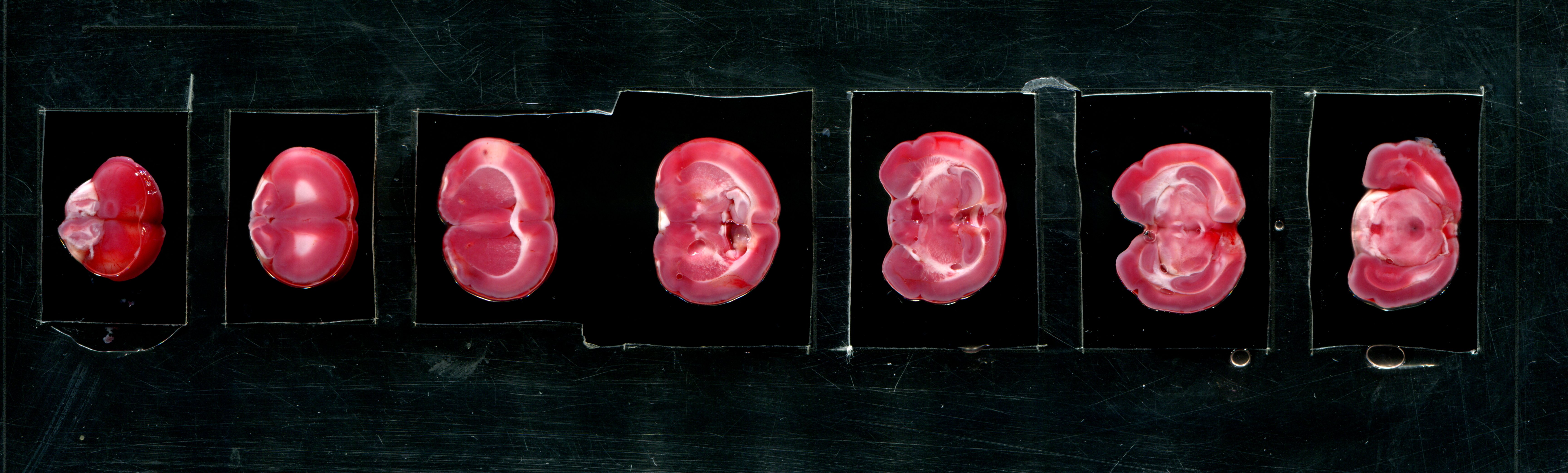

Supplement: S3 Archive — Coronal slices were stained using TTC. (ZIP) [file pone.0144659.s003.zip › G0401-2_GSNO_R24bis.jpg]

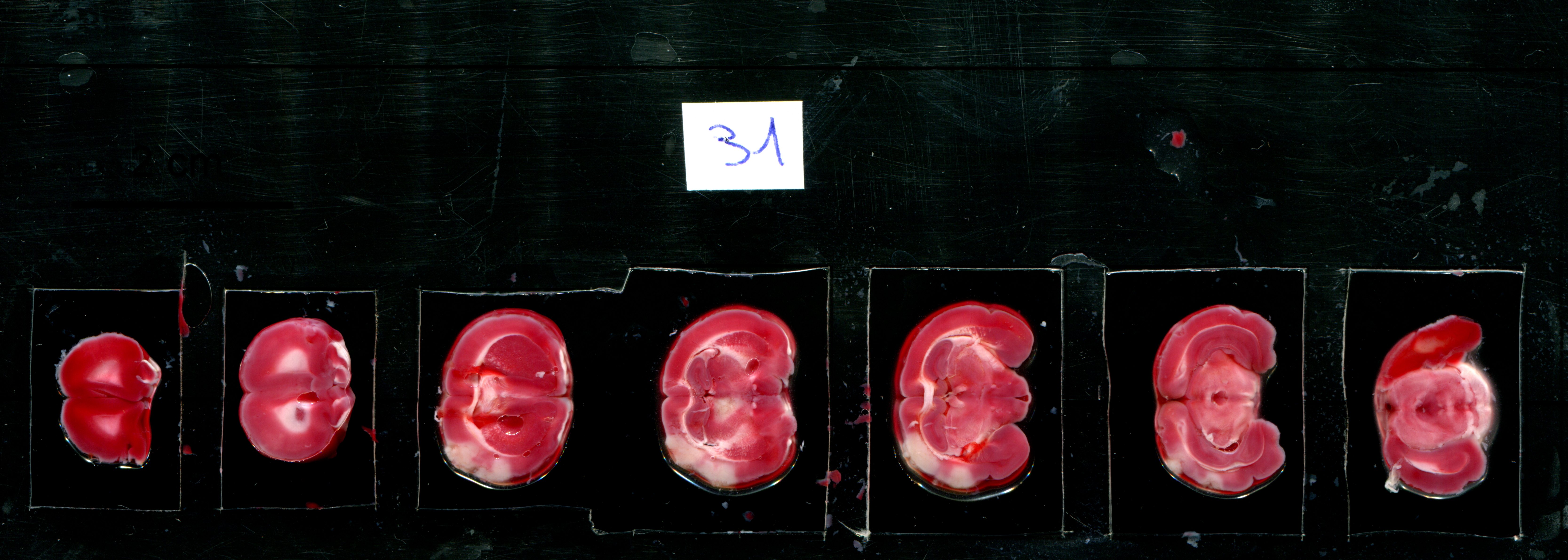

Supplement: S3 Archive — Coronal slices were stained using TTC. (ZIP) [file pone.0144659.s003.zip › G0401-2_GSNO_R31bis.jpg]

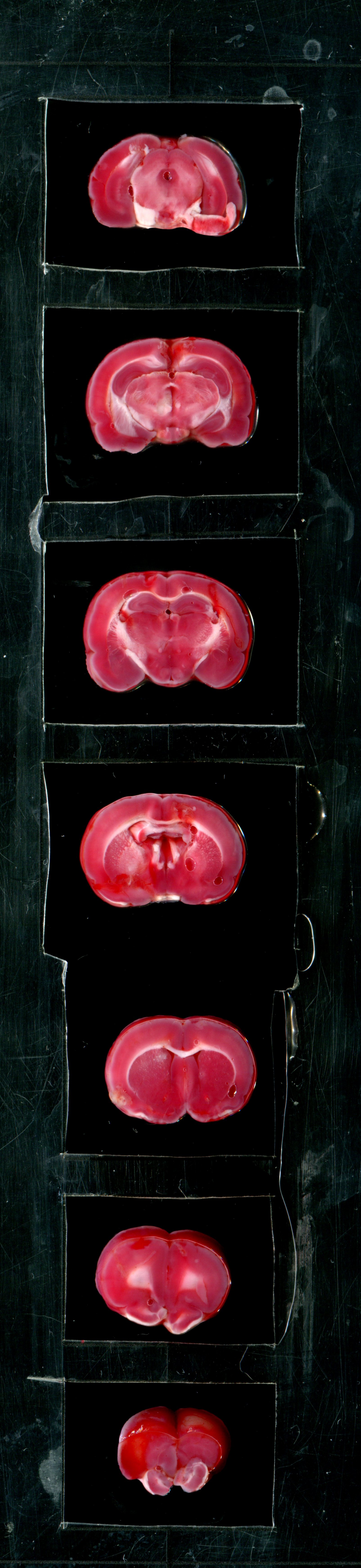

Supplement: S3 Archive — Coronal slices were stained using TTC. (ZIP) [file pone.0144659.s003.zip › G0401-2_GSNO_R33bis.jpg]

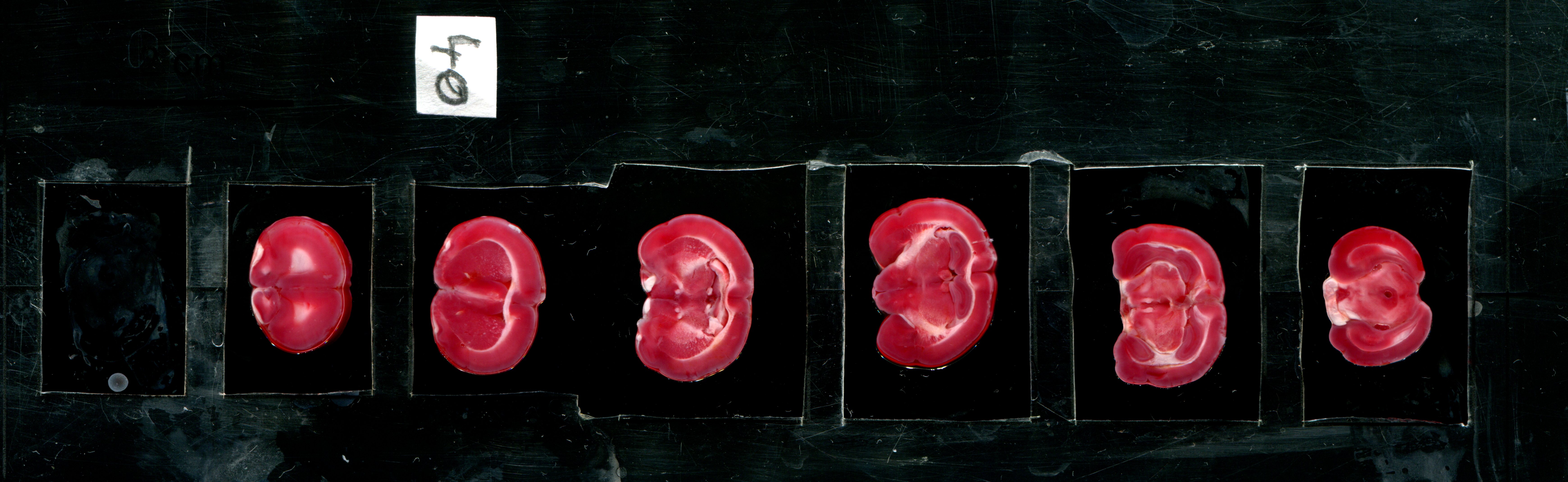

Supplement: S3 Archive — Coronal slices were stained using TTC. (ZIP) [file pone.0144659.s003.zip › G0401-2_GSNO_R40bis.jpg]

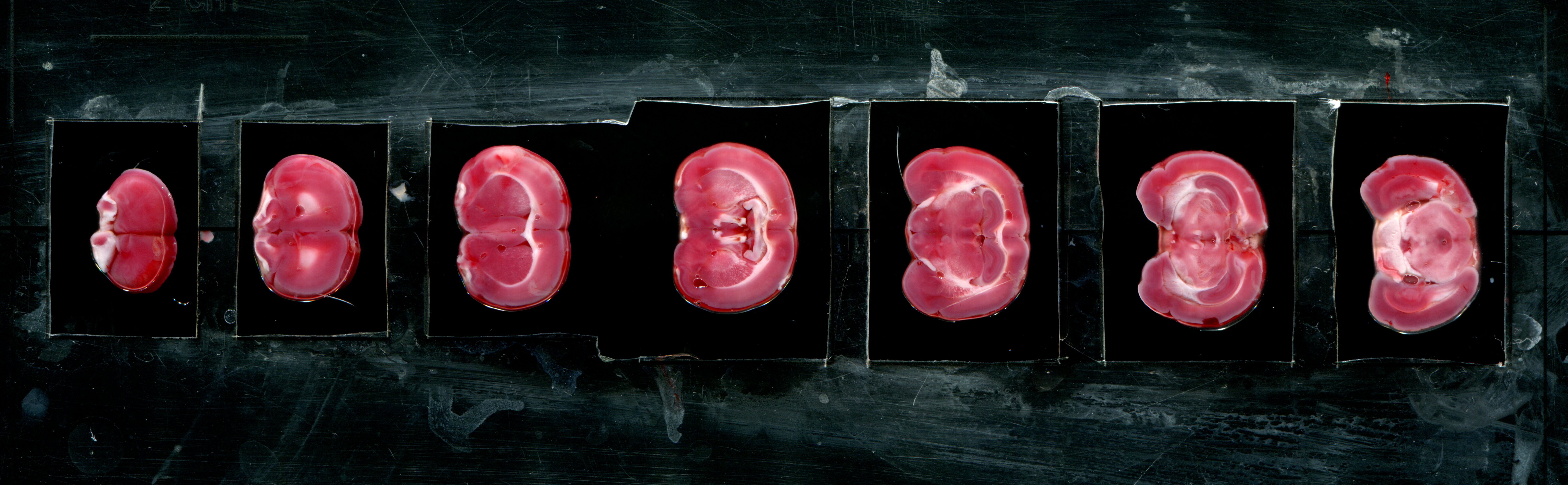

Supplement: S3 Archive — Coronal slices were stained using TTC. (ZIP) [file pone.0144659.s003.zip › G0401-2_GSNO_R47bis.jpg]

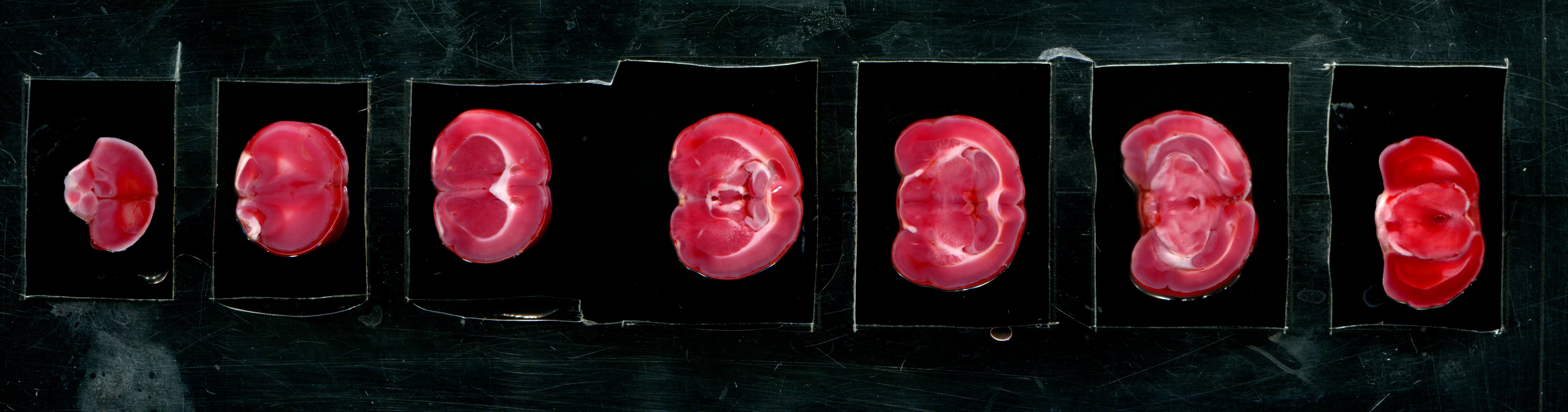

Supplement: S4 Archive — Coronal slices were stained using TTC. (ZIP) [file pone.0144659.s004.zip › G0401-2_GSNO_R3bis.jpg]

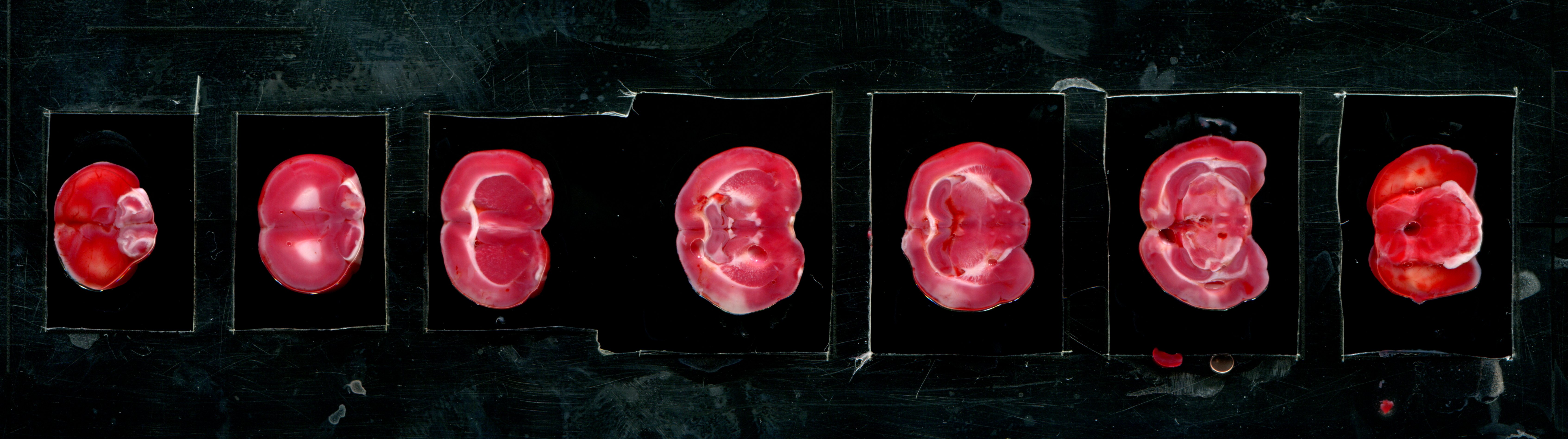

Supplement: S4 Archive — Coronal slices were stained using TTC. (ZIP) [file pone.0144659.s004.zip › G0401-2_GSNO_R10bis.jpg]

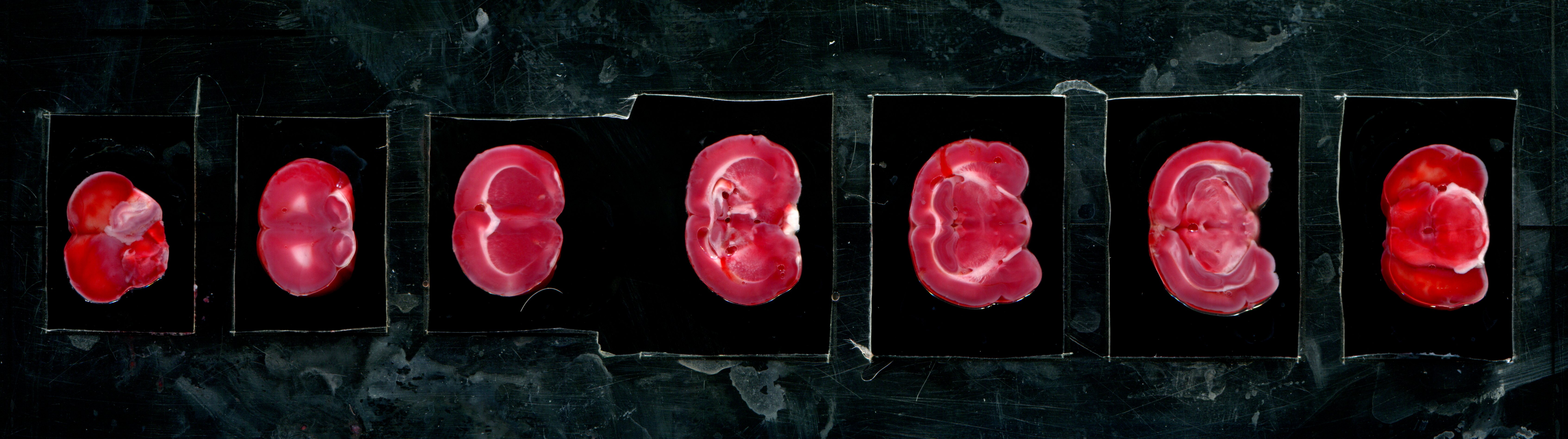

Supplement: S4 Archive — Coronal slices were stained using TTC. (ZIP) [file pone.0144659.s004.zip › G0401-2_GSNO_R15bis.jpg]

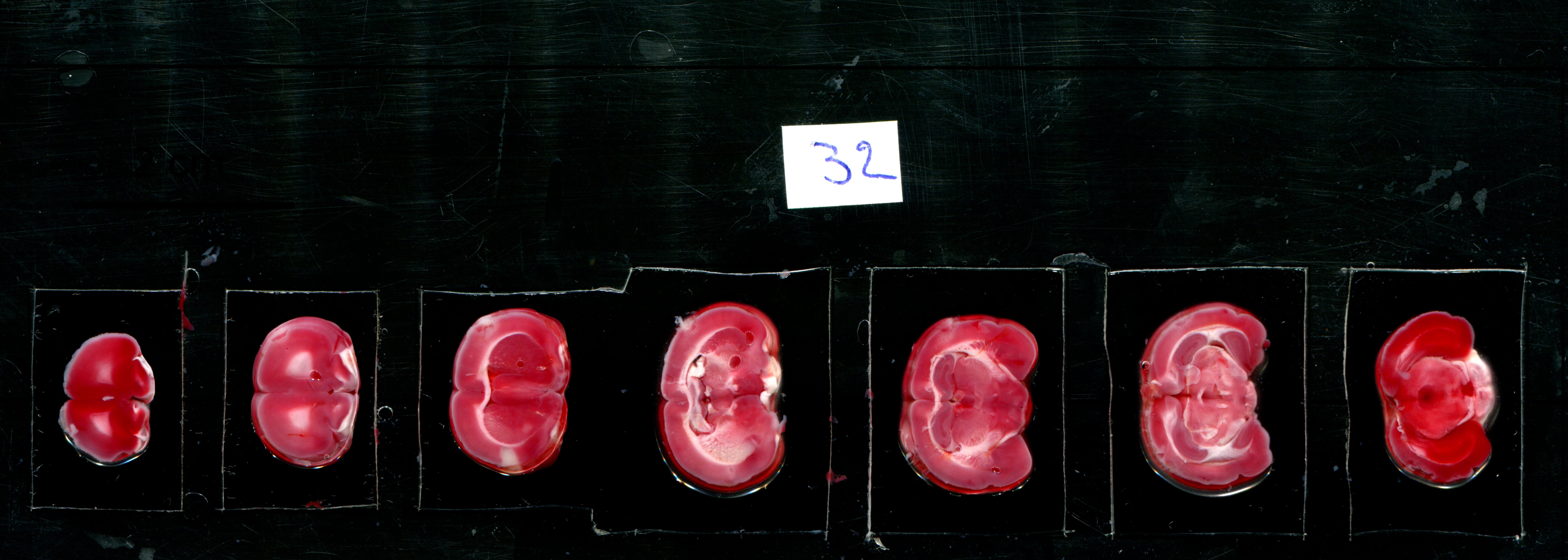

Supplement: S4 Archive — Coronal slices were stained using TTC. (ZIP) [file pone.0144659.s004.zip › G0401-2_GSNO_R32bis.jpg]

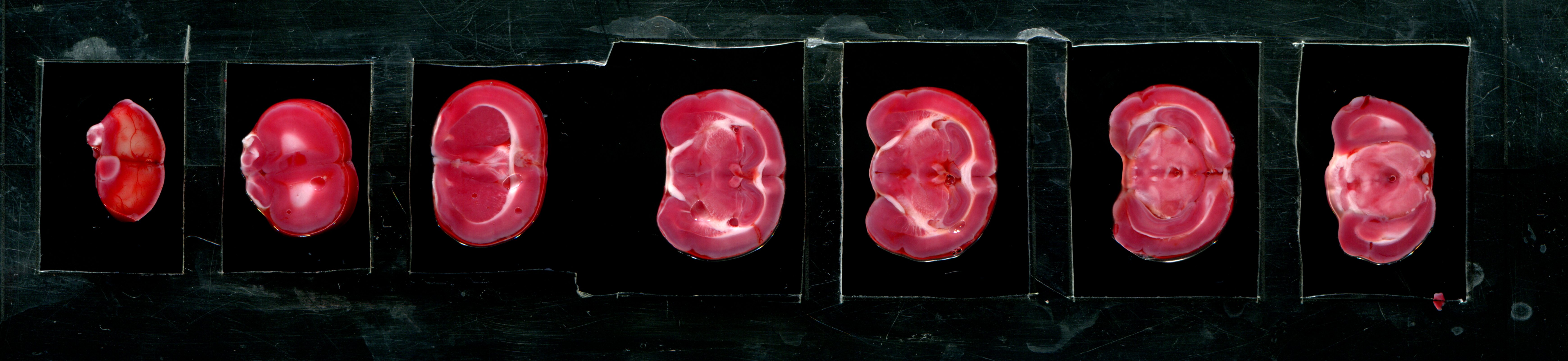

Supplement: S4 Archive — Coronal slices were stained using TTC. (ZIP) [file pone.0144659.s004.zip › G0401-2_GSNO_R36bis.jpg]

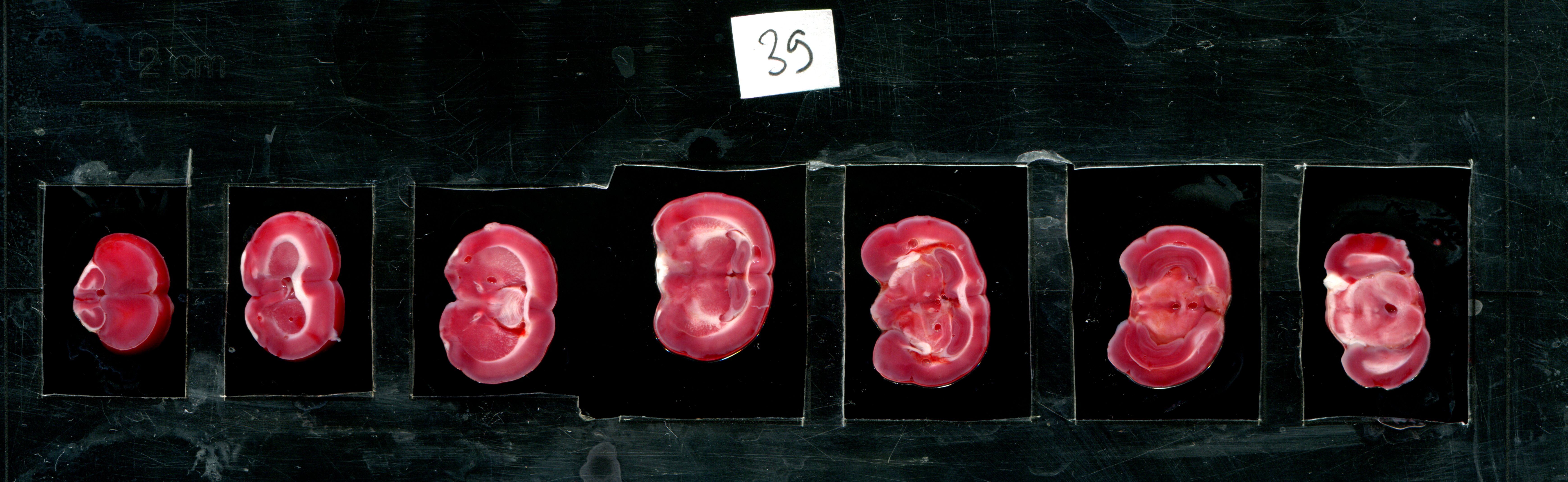

Supplement: S4 Archive — Coronal slices were stained using TTC. (ZIP) [file pone.0144659.s004.zip › G0401-2_GSNO_R39bis.jpg]
